# The Neighborhood Resources that Shape Children’s Well-Being: A Systematic Literature Review

**DOI:** 10.1007/s12187-026-10362-x

**Published:** 2026-04-13

**Authors:** Julia Steenwegen, Donna de Maat, Nicole Lucassen, Mathieu Gielen, Michelle Achterberg, Annabel Vreeker, Marije Kesselring, Joyce Weeland

**Affiliations:** 1 Youth and Family, ESSB, Erasmus University Rotterdam, Rotterdam, Netherlands; 2 Delft University of Technology, Delft, Netherlands; 3 Erasmus MC - Sophia Children’s Hospital, Rotterdam, Netherlands; 4 University of Applied Sciences Utrecht, Utrecht, Netherlands

**Keywords:** Neighborhood, Child well-being, Resources, Systematic review, Transdisciplinary research

## Abstract

Children’s well-being is partly shaped by the neighborhoods in which they grow up. While past research has focused predominantly on deficits in disadvantaged neighborhoods, this systematic review catalogues neighborhood resources that positively influence children’s well-being, adopting a strengths-based, interdisciplinary perspective. Drawing on 32 empirical studies across disciplines including psychology, sociology, health, and urban planning, this review explores which social, physical, and institutional neighborhood resources support children aged 6–12 years, as well as when and how. Findings reveal that social cohesion, perceived safety, peer relationships, and adult support enhance emotional and social well-being. Physical resources such as parks, green spaces, and walkable infrastructure foster physical activity and life satisfaction, while institutional supports, including libraries, after-school programs, and community centers, contribute to subjective well-being and academic engagement. The review highlights the importance of context: effects vary by age, gender, socioeconomic background, and cultural setting. Mediators such as lower parenting stress, neighborhood satisfaction, and family resilience elucidate the mechanisms through which neighborhood resources affect well-being. Notably, participatory and qualitative research underscores children’s own perspectives and agency, revealing valued but often overlooked informal neighborhood features. This review calls for equity-oriented, transdisciplinary approaches that integrate children's voices and foster neighborhood environments as active supports for well-being and development.

**Supplementary Information:**

The online version contains supplementary material available at https://doi.org/10.1007/s12187-026-10362-x.

Children's well-being is intricately linked to the environments in which they grow and develop (Evans, 2021; Maggi et al., 2010). Neighborhoods, which we define as the contexts that are relevant to the social and spatial environments in which children regularly engage (Humphrey, 2016; Sharkey & Faber, 2014), serve as one such critical environment. Neighborhoods shape the physical, social, and emotional environments of children (Christian et al., 2015; Leventhal & Dupéré, 2019; Mayne et al., 2021). Over recent years, there has been growing recognition that neighborhood characteristics—both positive and negative—can significantly influence children’s developmental trajectories, resilience and overall well-being (Beese et al., 2023; Boon et al., 2012; Li et al., 2017; Minh et al., 2017; Tappe et al., 2013). Historically, research on neighborhoods and child development has emphasized neighborhood deficits, highlighting how adverse conditions and lack of resources, such as environmental hazards, crime, poverty, limited social cohesion, and resource scarcity, hinder children’s development (Elías Alvarado, 2016; Choi et al., 2018; Jutte et al., 2015; Leventhal & Brooks-Gunn, 2000) and how these conditions in turn pose risks for delinquency, pathology and overall mental and physical health issues (Franzoi et al., 2024; Kim et al., 2019; Kohen et al., 2008; Wang et al., 2020). In contrast, much less is known about how neighborhoods might act as sources of support and resilience (Beese et al., 2023). This review adopts a strengths-based perspective (McDonell & Sianko, 2021), reflecting recent calls for equity-oriented and context-sensitive research. It increases our understanding of how neighborhoods can foster positive child development, and it identifies strategies to support community-based strengths. Importantly, this asset-based perspective highlights the resources in environments that are confronted with challenges and that are too often stigmatized.

Neighborhoods can offer essential resources for child well-being across multiple domains: social, institutional, and physical. Regarding *social resources*, research shows that strong social networks and community cohesion enhance social and emotional well-being in children, as children benefit from a sense of belonging and safety (Jespersen et al., 2021; Maguire-Jack & Marcal, 2022; Turkmani et al., 2023). Cohesive neighborhoods with a strong collective efficacy, meaning mutual trust and a shared willingness to act for the common good, have a positive effect on children’s adjustment (Yule et al., 2019). Regarding *institutional resources*, access to quality educational facilities and recreational areas foster social, cognitive and physical development (Poon et al., 2022). Moreover, after-school programs, community initiatives, and accessible meeting points can offer opportunities to offset possible threats to children's well-being and positively impact outcomes (McDaniel & Yarbrough, 2016). Finally, regarding *physical resources*, accessible, safe, well-maintained green spaces encourage physical activity and social interaction, building children’s resilience and cognitive, emotional and social skills (Liu & Green, 2023; McCormick, 2017; Pereira et al., 2024; Rubio-Cabañez, 2024; Sprague et al., 2022). Neighborhoods thus hold potential resources for children’s resilience and well-being, and children’s resilience is cofacilitated by their physical and social ecologies. Moreover, research shows that resilience depends on both the individual and the context, but that resilience on the level of the individual child is unsustainable when the systems surrounding the child (e.g., family, neighborhood) are not robust (Masten, 2018; Ungar & Theron, 2020). For example, during the COVID-19 pandemic, children living in more cohesive and safer neighborhoods fared better than others in terms of physical and mental health (Robinette et al., 2021).

Although there is research on various neighborhood domains, including the institutional, the physical and the social, it remains under-researched which specific resources in children’s neighborhoods contribute to their well-being. Research on this topic remains limited and scattered across various research disciplines and methodologies, with no studies offering an integrated perspective on potential resources across different domains. For example, some studies focus on social cohesion and collective efficacy, without considering how physical characteristics in the neighborhood affect child well-being (Maguire-Jack & Showalter, 2016). In addition, it is unclear how resources interrelate. Many studies focus narrowly on specific factors, such as crime or green spaces, without accounting for the interconnections between various neighborhood characteristics. For example, the benefits of a well-maintained playground may be limited if the area lacks safety or adult supervision, or the presence of green space may foster children’s physical activity, but its positive effects might be amplified, or limited, by other factors such as neighborhood safety, accessibility, or opportunities for social interaction. In this way, the physical and social dimensions of a neighborhood may interact in shaping children’s experiences, highlighting the importance of combined resources. In contrast to deficit-oriented reviews that primarily document risks or adverse neighborhood conditions (a.o. Metcalfe et al., 2011), this review deliberately adopts an asset-based perspective. Rather than assessing whether neighborhood characteristics are universally beneficial or harmful, the aim is to identify which neighborhood resources have been empirically associated with positive child well-being outcomes and can therefore be considered potential sources of well-being and resilience. By foregrounding resources linked to positive outcomes, this review seeks to inform research, policy, and practice on what may be meaningfully strengthened or leveraged within neighborhoods to support children’s well-being, particularly in contexts marked by structural disadvantage.

This review study systematically adresses these gaps by cataloguing findings across disciplines, from psychology to pedagogy, educational sciences, social work, sociology, health and urban planning, across methodologies, and across contexts (both urban and rural environments). The aim of this study is to provide a comprehensive overview of neighborhood characteristics that hold the potential to enhance child well-being and to explore the circumstances in which (when) and mechanisms (how) through which these resources exert their influence. By examining a diverse range of studies, this review primarily aims to identify neighborhood resources that support child well-being and amplify the assets within communities that can promote resilience, health, and well-being among children. In this review, we include both quantitative and qualitative studies and map potential mediators and moderators of the effects of neighborhood resources and child well-being.

We conceptualize well-being as the overall quality of children’s lives, including both objective well-being (e.g., material properties, risk behavior, and mental health) and subjective well-being (e.g., happiness, self-efficacy, and experienced quality of life) (Cho & Yu, 2020; Statham, & Chase, 2010). Acknowledging the multifaceted nature of neighborhoods impacting children’s well-being, we include physical, social, as well as institutional aspects of the neighborhood which can potentially enhance child well-being.

## Methods

This systematic review is preregistered on the Open Science Framework; see https://osf.io/bnq75?view_only=0b5ffc7c332040c3b4e4a04c1dc153b7. The aim of this review was to map which neighborhood factors are described in the literature and how these factors fit within the theoretical framework of resources for well-being, rather than to test the strength or consistency of empirical effects. This distinction justifies different inclusion criteria than those used in effect-focused systematic reviews.

Within this configurative review, the unit of analysis was the factor or construct, not the statistical effect. Studies reporting null findings were therefore not excluded on the basis of their results, but because they did not contribute to new or conceptually informative factors or relationships relevant to the framework. In this context, null findings do not contribute to cataloguing resources or advance theoretical elaboration or refinement and fall outside the scope of the review.

### Eligibility Criteria

Primary quantitative and qualitative empirical studies were considered if published in peer-reviewed journals and written in English. Papers without original data, review articles, unpublished manuscripts, protocol papers, methodological studies, book chapters, or conference proceedings were not eligible for inclusion. Gray literature was not searched, we deliberately restricted inclusion to empirical, peer-reviewed studies. This aligns with the aim of providing an evidence-based inventory of neighborhood factors contributing to children’s well-being and ensures that the review remains focused, transparent, and reproducible.

Acknowledging the dynamic nature of neighborhoods and their evolving influence on children's well-being, we have included empirical studies published from January 2005 to April 2025. This timeframe reflects key societal shifts, advances in research methodologies, and contemporary urban and social policy changes that shape children's neighborhood experiences today. Earlier studies may not capture critical developments such as increasing urbanization, digital transformation, and policy interventions aimed at fostering child-friendly communities. By focusing on the past two decades, this review ensures relevance to current neighborhood structures and child well-being frameworks, while cataloguing insights that are actionable for researchers, policymakers, and practitioners alike. The following eligibility criteria were applied:

For the population, we focused on children aged between six and 12 years. If papers covered populations younger than six or older than 12 but provided separate results for children between six and 12, papers were included and only the relevant results were taken into account.

We included multifaceted neighborhood factors and categorized those as (1) social neighborhood resources (e.g., cohesion, safety), (2) physical neighborhood resources (e.g., greenness, play areas), and (3) institutional neighborhood resources (e.g., libraries, public services, extracurricular activities). We included possible mediators and moderators to explore when, how, and for whom different neighborhood factors promote children’s well-being.

The outcome data had to reflect positive outcomes related to child well-being at the individual child level. In line with the asset-based aim of this review, we focused on studies that empirically demonstrated positive associations between neighborhood characteristics and child well-being outcomes. Studies reporting exclusively null or negative associations were not included. This decision was not based on assumptions about the prevalence or magnitude of neighborhood effects, but reflects the specific analytical focus of this review: identifying neighborhood resources that have shown potential to support children’s well-being and resilience. As such, this review should be read as complementary to deficit-oriented or risk-focused reviews, rather than as an exhaustive synthesis of all neighborhood effects.

### Information Sources

**Fig. 1:**
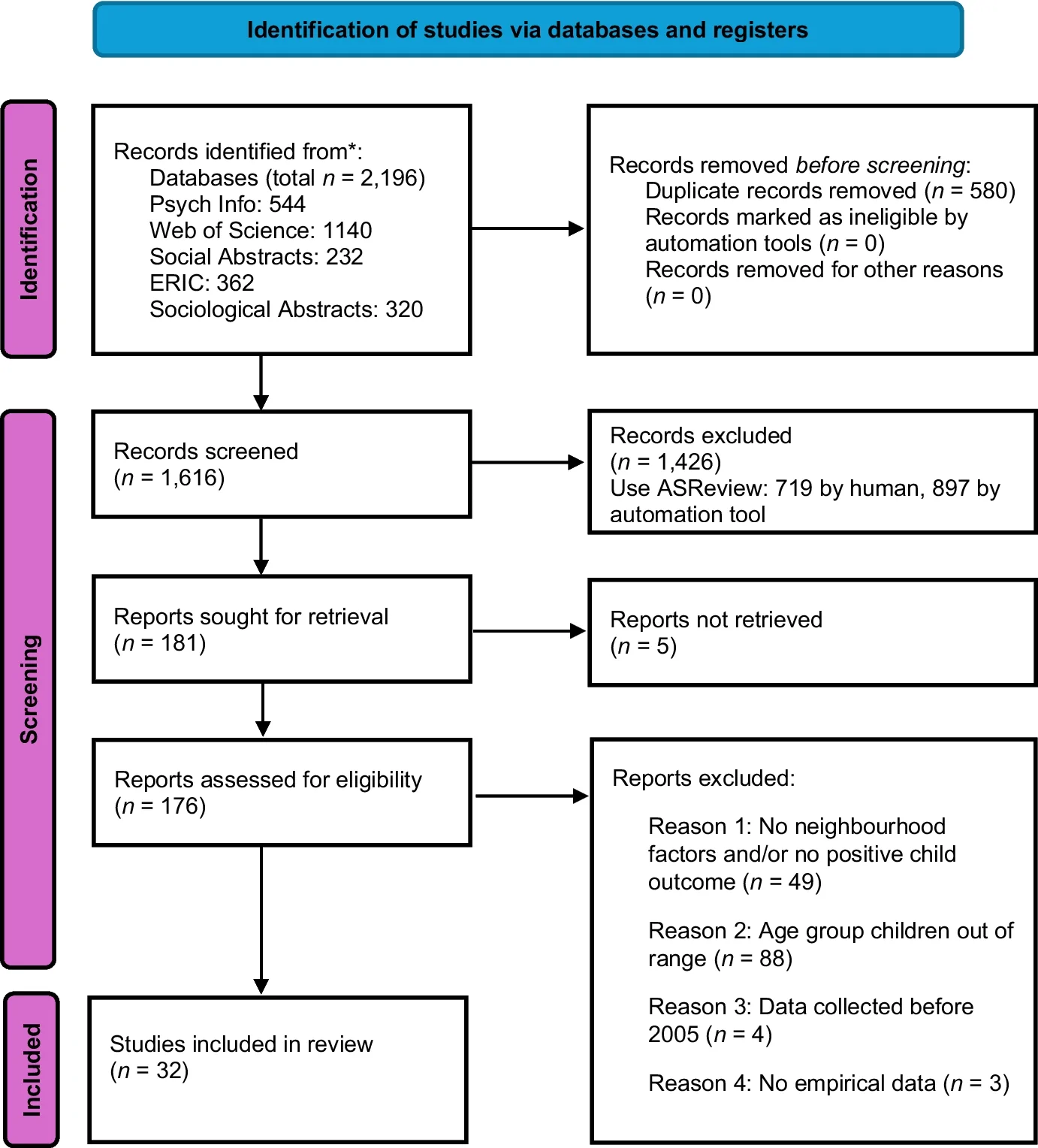
Prisma flow chart of included and excluded papers

Five electronic databases were used to reflect the interdisciplinary character of the research: Web of Science, PsycINFO, Sociological Abstracts, Social Abstract Services, and ERIC. The first search was conducted in **June 2024**, and a second search was conducted in **February 2025** to update the findings (Fig. 1).

### Search Strategy

The preregistered search strategy was tailored to each database. Filters included language (English) and peer-reviewed journal articles. Search terms were developed by an interdisciplinary team of colleagues from different fields such as public policy, sociology, psychology, educational science, built environment, design, health and pedagogy, to ensure inclusivity and to minimize over-reliance on a single discipline by limiting the use of excessive synonyms. We selected ‘well-being’ as a central term and included ‘belonging’ to capture sociological perspectives and acknowledge the importance of a sense of belonging in the neighborhood for children’s well-being (Roffey, 2013). Additionally, we included ‘flourishing’, ‘life satisfaction’, and ‘happiness’ as synonyms for subjective well-being based on the feedback from our interdisciplinary team. We focused on the neighborhood to highlight the importance of the physical space in which children live. We included the synonym ‘borough’ for reasons of inclusion. After a phase of explorative searches, we decided not to include synonyms such as ‘community’, ‘precinct’, ‘jurisdiction’ and ‘area’ due to their varied connotations. The search terms were deliberately broad with a combination of *well-being* (OR *wellbeing* OR *belonging* OR *flourishing* OR *life satisfaction* OR *happiness*) AND *child* (OR *children* OR *youth* OR *pupil*) AND (*neighborhood* OR *neighborhood* OR *borough*).

### Selection Process

Following the PRISMA Guidelines, first, the duplicates were removed using Endnote by one reviewer (J.S.), after which we made use of ASReview software (ASReview LAB developers, 2023) to screen the abstracts. This software makes use of researcher-in-the-loop learning in which the software regularly returns to the full list of titles to ensure avoidance of ‘trap formation’ (Sijtsma et al., 2021). The first 20 abstracts were screened by two reviewers simultaneously (J.S. and D.M.) to calibrate criteria. One reviewer (J.S.) continued to screen the abstracts using ASReview. The full text screening was primarily conducted by one reviewer (J.S., with 10% checked by a second reviewer (D.M.) and another 10% by a third reviewer (J.W.)) with disagreements resolved through team discussions. Interrater reliability was 92.5%. For full screening guidelines see Appendix A.

### Data Collection Process and Data Items

Data extraction was structured in an Excel worksheet, including; Authors, year of publication, country of data collection, Sample characteristics (sample size, children’s age, child sex, child education year level), Study design (qualitative, quantitative, mixed method, participative), Method (instruments, informants, Measured outcomes, Measured neighborhood characteristics, Main results (findings, moderators, mediators, effect estimates) and potential resources. One reviewer (J.S.) extracted the data, with a second reviewer (D.M.), and third reviewer (J.W.) cross-checking 20% of the articles.

### Strategy for Data Synthesis

One reviewer (J.S.) synthesized data independently, validated by the second reviewer (D.M.). Results were grouped based on social, institutional and physical factors addressed in the studies.

### Risk of Bias Assessment

For the qualitative studies, we used the CASP checklist for qualitative studies (Long et al., 2020). The eight included qualitative studies that scored well on research rigor overall. It should be noted though that only two papers included a reflection on the researchers’ positionality. See Appendix B for the checklists of all included papers.

For the quantitative studies, risk of bias was assessed using the Appraisal tool for Cross-Sectional Studies (AXIS) (Downes et al., 2006). We added one item derived from the NIH Quality Assessment Tool for Observational Cohort and Cross-Sectional Studies, namely whether loss to follow-up after baseline was 20% or less. To support this time-consuming and structured extraction task, ChatGPT was used as an assistive tool to populate preliminary AXIS item responses in tabular form. Importantly, ChatGPT outputs were not treated as final judgements but as an initial draft comparable to a first-pass extraction that might otherwise be conducted by a trained research assistant. The first two bias tables were compared with a fully manual assessment completed by the lead reviewer (J.S.), after which the prompt was refined accordingly. In addition, 10% of the tables were double-coded by ChatGPT and an independent reviewer (D.M.). Based on this comparison, response-rate information was manually verified and corrected where needed, as ChatGPT sometimes incorrectly indicated that no information was available. All bias assessments were reviewed, edited, and finalized by the research team, who retain full responsibility for the methodological judgements. See Appendix completed bias tables. Given emerging evidence that large language models can produce plausible but insufficiently reliable research quality evaluations when used without human oversight, ChatGPT was used strictly as a workflow aid rather than an autonomous evaluator (Thelwall, 2024).The bias check showed that, overall, the included studies had a sound methodological design and appropriate reporting quality. The evidence base is dominated by cross-sectional observational studies, supplemented by two longitudinal studies (Fava et al., 2022; Zhou et al., 2021) and one quasi-experimental study (Hong et al., 2025). While sample sizes are often medium to large, formal sample size justifications are rare, and non-response bias is frequently insufficiently addressed. This makes it unclear whether studies had sufficient power to detect associations between neighborhood resources and well-being. Overall, the strength of evidence for associations between neighborhood resources and child well-being is moderate for associative relationships but low for causal effects. Additionally, the representativeness and potential for selection bias were difficult to assess, due to the absence of information on participant selection, non-responders, and response rates in multiple studies.

## Results

We included a total of 32 studies, of which 23 studies were quantitative, utilizing surveys, structured assessments, and large-scale datasets to measure neighborhood influences on child well-being. Of those qualitative studies, 20 had a cross-sectional, two a longitudinal and one a quasi-experimental design. Eight studies were qualitative, capturing children's lived experiences through interviews, focus groups, and participatory mapping. Of those qualitative studies, six studies employed Participatory Action Research (PAR), directly involving children in the research process to co-produce knowledge about their neighborhood environments. In 25 studies, children were the primary informants, either self-reporting well-being or participating in qualitative explorations. In the seven remaining cases, parents, teachers, or community members provided insights into neighborhood impacts on child development studies.

The studies were conducted in more than 40 different countries. Ten studies were solely conducted in the US. Furthermore, by bringing together findings from various disciplines, the umbrella of ‘well-being’ as outcome harbors a diverse set of positive outcomes ranging from flourishing to academic adjustment. The most frequently measured outcome was subjective well-being (8 studies) and health or health-related quality of life (6 studies). An overview of the information on the studies, samples, and methods can be found in Table 1.

**Table 1:** Overview of the methodology of included papers

Study	Journal	Country	Sample size Age in years	Method	Instruments	Informants
Alfaro et al., 2024	Journal for Child and Family Studies	Chile	1,065 10–12	Quantitative	ISCWeB (International Survey of Children's Well-Being) from 2018 SLSS (Students’ Life Satisfaction Scale)	Children
Ashiabi, 2018	International Journal of Child, Youth and Family Studies	US	27,752 6–11	Quantitative	NSCH (National Survey of Children’s Health) from 2007	Parents
Barnhart et al., 2022	Children	US	18,396 6–11	Quantitative	NSCH (National Survey of Children’s Health) from combined 2018 and 2019	Parents
Corominas et al., 2021	Children and Youth Services Review	Spain	3,962 10–12	Quantitative	Children’s Worlds Questionnaire CW-SWBS (Children's Worlds Subjective Well-Being Scale) , based on the Students’ Life Satisfaction Scale	Children
den Besten, 2010	Childhood	Germany, France	233 10–13	Qualitative	Subjective maps	Children
Ditzel et al., 2023	Child Indicators Research	Israel, Chile	4,942 10 and 12	Quantitative	Children’s Worlds Survey CW-SWBS (Children’s Worlds Subjective Well-Being Scale), based on the Students’ Life Satisfaction Scale	Children
El-Dardiry et al., 2012	Child Indicators Research	Greece	800 8–12	Quantitative	KIDSCREEN-52	Children
Eriksson & Dahlblom, 2020	Social Science & Medicine	Sweden	41 11,5	Qualitative	Participant Action Research	Children
Fava et al., 2022	Maternal and Child Health Journal	US	4,689 3, 5 and 9	Quantitative Longitudinal	Fragile Families and Child Well- Being Study	Parents
Forrester et al., 2024	Child and Adolescent Social Work Journal	US	69 9,3	Quantitative	Children’s Worlds Survey	Children
Gadermann et al., 2016	Journal of Happiness Studies	Canada	5,026 9–10	Quantitative	Middle Years Development Instrument	Children
González-Carrasco et al., 2019	Applied Research in Quality Life	Israel, Spain, Algeria, South Africa	9,262 10–12	Quantitative	SLSS (Student’s Life Satisfaction Scale) BMSLSS (Brief Multidimensional Student’s Life Satisfaction Scale)	Children
Hong et al., 2025	Journal for Urban Health	US	398 3–11	Quasi experimental (intervention)	KINDL- questionnaire for health-related quality of life	Parents
Horgan et al., 2023	Irish Journal of Sociology	Ireland	39 11,5	Qualitative	Participant action research	Children
Kandasamy et al., 2018	Journal of Developmental & Behavioral Pediatrics	US	59,362 6–17	Quantitative	NSCH (National Survey of Children's Health)	Parents
Lawler et al., 2018	Children and Youth Services Review	US, South-Korea	1,775 10–12	Quantitative	Children's Worlds Survey	Children
Lee & Yoo, 2015	Child Indicators Research	Algeria, Brazil, Chile, England, Israel, Romania, South Africa, South Korea, Spain, Uganda, US	12,077 12	Quantitative	ISCWeB (pilot study of the International Survey of Children’s Well-Being) 2011–2012 GDSI (General Domain Satisfaction Index)	Children
Liu et al., 2018	Health and Place	US	49,513,974 6–17	Quantitative	NSCH (National Survey of Children's Health)	Parents
Minguez, 2020	Journal of Happiness Studies	Germany, UK, Spain, Norway,	4812 9–12	Quantitative	ISCWeB (International Survey of Children’s Well-Being)	Children
Newland et al., 2015	Child Indicators Research	US	1286 10,7	Quantitative	Children's Worlds Survey	Children
Oberle et al., 2011	Journal of Youth and Adolescence	Canada	1,402 11,5	Quantitative	SWLS-C (Satisfaction With Life Scale for Children) California Healthy Kids Survey subscales for neighborhood and parental support Resiliency Inventory subscales for positive peer relationships and optimism	Children
Oberle et al., 2014	Canadian Journal of School Psychology	Canada	3,026 10,8	Quantitative	MDI (Middle Years Development Instrument)	Children
Rogers, 2012	Child Indicators Research	Ireland	132 7–12	Qualitative	Participant Action Research	Children
Shortt & Ross, 2021	Social Science and Medicine	Scotland	15 11,5	Qualitative	Participant Action Research	Children
Turner et al., 2006	Health Education	Scotland	67 8–14	Qualitative	Interviews Focus Groups	Children
Ucci et al., 2022	International Journal of Environmental Research and Health	UK	2–12	Qualitative	Interviews Online questionnaire	Parents
van den Berg et al., 2020	Journal of Transport and Health	Netherlands	660 7–12	Quantitative	Instrument developed for the study	Children
Wang et al., 2023	Child Indicators Research	Albania, Algeria, Bangladesh, Belgium, Brazil, Chile, Croatia, England, Estonia, Finland, France, Germany, Greece, Hong Kong, Hungary, India, Indonesia, Israel, Italy, Malaysia, Malta, Namibia, Nepal, Norway, Poland, Romania, Russia, South Africa, South Korea, Spain, Sri Lanka, Switzerland, Taiwan, Vietnam, and Wales	93,344 10,9	Quantitative	ISCWeB (International Survey of Children’s Well-Being)	Children
Witherspoon et al., 2016	American Journal of Community Psychology	US	227 8,6	Quantitative	Survey adapted from existing scales	Children
Witten et al., 2019	Social and cultural Geography	New Zealand	253 9–12	Qualitative	Interviews GPS data Focus groups	Children
Wu et al., 2010	Quality of Life Research	Canada	3,421 10–11	Quantitative	EQ-5DY Canadian English child version	Children
Zhou et al., 2021	Applied Research in Quality of Life	China	519 5–9	Quantitative Longitudinal	Questionnaire from the Community Survey of the Project on Human Development in Chicago Neighborhoods for neighborhood cohesion and neighborhood tie KINDL-R for Health-Related Quality of Life Social indicators of well-being for subjective well-being	Children

### Neighborhood Resources, Moderation and Mediation Effects

This review systematically catalogues which neighborhood characteristics may positively contribute to children's well-being, focusing on social, physical, and institutional resources, when and how. In 27 of the 32 studies, evidence was found for social resources, in 20 studies for physical resources, and in 14 studies for institutional resources. Identified neighborhood social resources were: (experienced) safety and surveillance, social cohesion, spaces for social interaction, social ties or capital, (parental) social support, peer belonging and friends, satisfaction with the people or atmosphere, and collective efficacy. Identified physical resources were: (good) infrastructure/street connectivity, sidewalks or walking paths, parks or playgrounds, satisfaction with outdoor spaces, outdoor space for physical movement and play, outdoor spaces for family and friends, (improved) green spaces, sport facilities/ indoor activity spaces, and quality of bicycle infrastructure. Finally, identified institutional resources were: recreation/community centers or (youth) clubs, library or bookmobile, sports, access to a variety of foods, presence of amenities, neighborhood quality, public transport, access to free or cheap activities, community roles (teachers, police), after-school provisions and sports facilities, and neighborhood satisfaction/service (Table 2).

**Table 2:** Overview of the findings of included studies

Study	Child outcome	Social resources	Physical resources	Institutional resources	Significant findings and mechanisms	Effect	Moderators/mediators
Alfaro et al., 2024	Life satisfaction	Feelings of safety	Urban infrastructure and livability conditions		Feelings of safety and urban infrastructure and livability conditions were positively associated with life satisfaction	β = 0.27*	-
Ashiabi, 2018	Social adjustment School engagement	Neighborhood safety Neighborhood social cohesion	Sidewalks and walking paths Parks and playgrounds	Recreation, community centers, and clubs Library and bookmobile	Presence of amenities was positively associated with social adjustment and school engagement Neighborhood safety and social cohesion were positively associated with social adjustment Associations were mediated by parenting stress, parental well-being, and family processes	Amenities à social adjustment: β = 0.03** Amenities à school engagement: β = 0.02* Social cohesion à social adjustment: β = 0.03* Safety à social adjustment: = β = 0.03*	Moderator: Age Mediators: Parenting stress Parental well-being Family processes
Barnhart et al., 2022	Flourishing	Social cohesion			Social cohesion was positively associated with flourishing directly and indirectly through family resilience	Neighborhood social cohesion à flourishing: β = 0.09** Social cohesion à family resilience: β = 0.27** Family resilience àflourishing: β = 0.29**	Mediator: Family resilience
Corominas et al., 2021	Subjective well-being		Outdoor play time Safe play outdoor spaces		Outdoor play time and safe play outdoor spaces were positively associated with subjective well-being	-	-
den Besten, 2010	Sense of belonging	Spaces for social interaction		Youth club	Spaces for social interaction were especially important for disadvantaged youth	N/A	N/A
Ditzel et al., 2023	Subjective well-being	Neighborhood safety	Areas to play		Neighborhood safety and areas to play were positively associated with children's subjective well-being, which was partially mediated by "satisfaction with the neighborhood" Significant effects observed only in Chile Older children showed a stronger association of neighborhood satisfaction with subjective well-being	-	Moderators: Age Country Mediator: Satisfaction with the neighborhood
El-Dardiry et al., 2012	Health-related quality of life	Social capital Parental social support			Neighborhood social capital and parental social support were both independently and positively associated with child self-reported health-related quality of life	Social capital à child self-reported health-related quality of life: β range = 0.03 - 0.20 Parental social support à child self-reported health-related quality of life: β range = 0.07–0.19	-
Eriksson & Dahlblom, 2020	Well-being	Spaces for social interaction	Areas for play	Sports	Unequal distribution in access to spaces leads to unequal well-being in neighborhood	N/A	N/A
Fava et al., 2022	Well-being	Collective efficacy	-	-	More positive maternal perception of neighborhood collective efficacy at age 3 predicted better children’s well-being at age 9 Maternal well-being partially mediated the relationship between maternal perception of neighborhood collective efficacy and child well-being	β = 0.21***	Mediator: Maternal well-being
Forrester et al., 2024	Life satisfaction Core affect	Neighborhood quality			Neighborhood quality was positively associated with children’s life satisfaction and core affect	Neighborhood quality à life satisfaction: ΔR^2^ = 0.18; β = 0.43 Neighborhood quality à core affect: ΔR^2^ = 0.08; β = 0.28	-
Gadermann et al., 2016	Life satisfaction perceived health	Connectedness with adults Peer belonging		Sports	Peer belonging and connectedness with adults (school) were positively related to children’s life satisfaction Peer belonging, connectedness with adults (school, neighborhood), and sports were positively associated with children’s perceived health	Peer belonging à life satisfaction PRATT (*d*) = .39*** Connectedness with adults at school à life satisfaction PRATT (*d*) = .12* Peer belonging à perceived health PRATT (*d*) = .36*** Connectedness with adults at school à perceived health PRATT (*d*) = .11*** Connectedness with adults in the neighborhood à perceived health PRATT (*d*) = .09*** Sports à perceived health: PRATT (*d*) = .05***	-
González-Carrasco et al., 2019	Subjective well-being (including satisfaction with safety and satisfaction with life as a whole)	Satisfaction with the people	Areas for play Satisfaction with outdoor spaces		Satisfaction with the people, areas for play, and satisfaction with outdoor spaces were related to children’s perceptions of safety in the nearby area In turn, perceptions of safety in the nearby area were associated with higher levels of subjective well-being (satisfaction with life as a whole)	Satisfaction with the people à Perceptions of safety in the nearby area: OR = 1.11* Areas for play à Perceptions of safety in the nearby area: OR = 1.29* Satisfaction outdoor spaces à Perceptions of safety in the nearby area: OR = 1.06* Perceptions of safety in the nearby area à satisfaction with life as a whole: OR = 1.08*	Moderators: Country Sex
Hong et al., 2025	Health-related quality of life		Park renovation		Physical well-being increased after park renovation, but not for overall well-being, emotional well-being, self-esteem, family relationship, friend relationships, or school functioning. Benefit of park quality improvement on physical well-being was stronger in girls than boys and among children from the lowest socio-economic status, specifically those living with parents who were single/divorced/widowed, less educated, not employed, or had an annual household income < $20,000	Physical well-being increased in the intervention group (Δ = 7.25, *SE* = 2.10***), but not the control	Moderators: Child sex Parental education Parental employment Households income Parental partner status
Horgan et al., 2023	Well-being	Spaces for social interaction Local convenience store	Areas for play	Access to a variety of foods	Accessibility of spaces is important to facilitate interaction with peers	N/A	N/A
Kandasamy et al., 2018	Flourishing	Safety Social support		Presence of amenities	Safety, social support, and presence of amenities were positively associated with children’s flourishing Neighborhood factors, together with other factors, explain the relationship between certain sociodemographic factors (such as poverty and family structure) and child flourishing	Safety: OR = 1.14* Social support: OR = 1.09* Presence of amenities: OR = 1.09*	-
Lawler et al., 2018	Subjective well-being	Satisfaction with the people	Areas to play Areas safe to walk Satisfaction with outdoor spaces Satisfaction with area in general	Neighborhood quality	Neighborhood quality was positively associated with children's subjective well-being in South Korea, but not in the United States	β _South-Korea_ = 0.20⁎⁎⁎	Moderator: Country
Lee & Yoo, 2015	Subjective well-being	Safety	Areas to play		Safety and areas to play were positively associated with subjective well-being	Safety and areas to play together explain 7% of the variation in children’s subjective well-being across countries Safety à children’s subjective well-being: β = 0.19*** Areas to play à children’s subjective well-being: β = 0.16***	-
Liu et al., 2018	Health Flourishing		Parks Sidewalks	Recreation center Library Public transport	Children in neighborhoods with high assets and low disorganization (Class 1) have better health outcomes and higher levels of flourishing, compared to those in other classes	Not reported	-
Minguez, 2020	Subjective well-being	Safe play areas	Safe walking areas		Neighborhood safety (safe play and walking areas) was positively associated with children’s subjective well-being	*r* partial = .15***	-
Newland et al., 2015	Subjective well-being			Neighborhood quality	Neighborhood quality was positively associated with children’s subjective well-being (including life satisfaction, mental health, and self-image) Neighborhood quality was positively associated over time with children’s subjective well-being (including mental health and self-image)	Correlations: Life satisfaction: *r* = .51 *** Mental health: *r* = .56*** Self-image:* r* = .55 *** Regression analyses: Mental health: β = 0.19*** Self-image: β = 0.18***	
Oberle et al., 2011	Life satisfaction	Social support not-related adults	-	-	Social support from not-related adults was positively associated with children’s life satisfaction	Individual level: γ = .05* School level: γ = .60*** A one-point increase in average perceived social support was associated with a .05 (individual level) and .60 (school level) increase in life satisfaction	-
Oberle et al., 2014	Emotional well-being	Social support Not-related adults			Social support from not-related adults was positively associated with children’s emotional well-being	β = 0.11*** PRATT (*d*) = .10 Social support from not-related adults explained 10% of the total variance in emotional well-being	-
Rogers, 2012	Well-being perspective on the neighborhood	Neighborhood friends	Outdoor space for physical movement and play		Children are active users of their neighborhoods, with valuable local knowledge and unique insights. Friendships and opportunities for active play are central to their well-being, with ‘space’ and ‘friends’ being what they value most about their neighborhood	N/A	N/A
Shortt & Ross, 2021	Health and well-being	Visible responsible adults Support for substance misuse issues	Parks and open spaces Outdoor spaces for family and friends Improved green spaces	Access to free or cheap activities Community roles (teachers, police)	Children pointed towards access to family and friends, lack of littering and safety as important to their well-being. They also recognized how stigma and disadvantage shape their experiences. The children recommended better access to affordable activities, improved public spaces, more support (not stigma), and greater inclusion in local decision making	N/A	N/A
Turner et al., 2006	Perceptions of community	Being near friends Important adults	Parks Sport facilities	Local clubs and organized activities	Support from trusted people	N/A	N/A
Ucci et al., 2022	Health and well-being	Social support	Green spaces Hyperlocal play spaces	Essential services and amenities within walking distance Indoor activity spaces After-school provisions and sports facilities	Overcrowding and lack of outdoor space can exacerbate each other's effects on well-being, while community connection can improve feelings of safety and belonging	N/A	N/A
van den Berg et al., 2020	Satisfaction with transport Mood	Parental safety perception Social cohesion	Street connectivity Quality of bicycle and walking infrastructure	-	Social cohesion, street connectivity, and quality of bicycle and walking infrastructure were associated with parental safety perception Parental safety perception was positively associated with children’s satisfaction with transport, which in turn, was positively associated with children’s mood	Social cohesion à Parental safety perception: β = 0.15** Street connectivity à Parental safety perception: β = 0.19** Quality of bicycle and walking infrastructure à Parental safety perception: β = 0.18** Parental safety perception à satisfaction with transport: β = 0.13** Satisfaction with transport à mood: β = 0.47**	-
Wang et al., 2023	Subjective well-being	Neighborhood quality (kind adults, listening adults, helpful people, feeling safe)	Neighborhood quality (play areas)	Neighborhood quality (opportunities for community participation)	Neighborhood quality was positively associated with children’s subjective well-being Out-of-school activities (watching TV, playing sports / doing exercise, relaxing with family, playing / time outside, using social media, and playing electronic games) mediated the association between neighborhood quality and children’s subjective well-being	*r* = .30**	Moderator: Friendship quality Mediator: Out-of-school activities
Witherspoon et al., 2016	Academic Self-Efficacy	Positive neighborhood perceptions (friendliness, safety, and neighbors interest in education)	-	-	Positive neighborhood perceptions were positively associated with children’s academic self-efficacy directly and indirectly via racial pride	Positive neighborhood perceptions à Academic self-efficacy: β = 0.23* Positive neighborhood perceptions à Racial pride: β = 0.58** Racial pride à Academic self-efficacy: β = 0.44**	Mediator: Racial pride
Witten et al., 2019	Well-being and belonging	Caring neighbors and friends Neighborhood surveillance	Footpaths Parks Playgrounds Spaces for diverse play	-	Independence afforded by parents, presence of older youth, visual markers of the sex industry influence children's mobility, agency, and sense of safety and belonging in their neighborhoods	N/A	N/A
Wu et al., 2010	Health-related quality of life	-	Neighborhood sidewalks/parks	Neighborhood satisfaction/service	Children residing in neighborhood characterized as providing good satisfaction and facilities, and having sidewalks and parks reported higher Health-related quality of life	Neighborhood satisfaction/services: Lowest vs. highest third (95% CI quality of life): 76.9–79.4 vs. 80.8- 82.9 Neighborhood sidewalks/parks: Lowest vs. highest third (95% CI quality of life): 0.8- 0.9 vs. 0.9–0.9	-
Zhou et al., 2021	Health-related quality of Life Well-being	Neighborhood cohesion Neighborhood tie	-	-	The neighborhood environment mediated the association between family socio-economic status and children’s well-being	Neighborhood environment à children’s well-being outcomes: β = 0.49**	-

Only significant resources and results are reported. *p < .05, ** *p* < .01, ***p < .001; *N/A* not applicable, as it concerns a qualitative study

The strength of the association between neighborhood resources and child outcomes varied considerably across quantitative studies, ranging from very small *β* = 0.02 (Ashiabi, 2018 on amenities on school engagement) to large *β* = 0.60 (Oberle et al., 2011 on school-level social support on life satisfaction). On average, most studies found small to moderate effect sizes. Overall, for several neighborhood resources evidence was found in multiple studies, specifically for resources related to neighborhood quality, including infrastructure and livability (Alfaro et al., 2024; Forrester et al., 2024; Lawler et al., 2018; Wang et al., 2023). But also for neighborhood safety, social cohesion, connectedness and supportive social relationships, including parental support, peer belonging, and connections with adults outside the family. For some neighborhood resources inconsistent findings were shown across studies, for example, access to play and outdoor spaces. While studies such as Corominas et al. (2021), Lee and Yoo (2015), and Minguez (2020) found positive associations with child well-being, Horgan et al. (2023) and Ditzel et al. (2023) did not. Studies also indicate associations are not universal but are moderated by children’s age (Ashiabi, 2018; Ditzel et al., 2023); sex (Hong et al., 2025); country/cultural context (Ditzel et al., 2023; González-Carrasco et al., 2019; Lawler et al., 2018); friendship quality (Wang et al., 2023); socio-economic factors such as parental education, employment, and household income (González-Carrasco et al., 2019; Hong et al., 2025); and parental partner status (Hong et al., 2025). For example, Lawler et al. (2018) found that neighborhood quality was positively associated with children’s subjective well-being in South Korea, but not the United States, possibly shaped by cultural, social, or urban planning differences. In Hong et al. (2025), park renovations increased children’s physical well-being but had no measurable impact on overall, emotional, or social well-being. The benefits of neighborhood interventions may depend on the specific domain of well-being and local conditions. In Ditzel et al. (2023), neighborhood satisfaction was more strongly linked to subjective well-being in older children than in younger children, possibly indicating that developmental stage influences how children interact with and benefit from their neighborhood environment.

Moreover, the papers reported different mechanisms (mediators) through which neighborhood resources affect child outcomes, specifically parental well-being (Ashiabi, 2018; Fava et al., 2022); family processes (Ashiabi, 2018); family resilience (Barnhart et al., 2022); neighborhood satisfaction (Ditzel et al., 2023); out-of-school activities (Wang et al., 2023); racial pride (Witherspoon et al., 2016); and the overall neighborhood environment. (Zhou et al., 2021). For instance, parent-related mechanisms were found in Ashiabi (2018) and Fava et al. (2022) where parental well-being acted as a mediator between (perceived) neighborhood safety and collective efficacy and children’s well-being. Barnhart et al. (2022) found that neighborhood social cohesion increased children’s flourishing both directly and indirectly through enhanced family resilience. Children’s own perceptions and behaviors can also serve as mediators. For example, Ditzel et al. (2023) showed that neighborhood safety and areas to play were associated with children’s subjective well-being through their satisfaction with the neighborhood. Finally, broader structural conditions such as the overall neighborhood environment were highlighted by Zhou et al. (2021), mediating the relationship be**t**ween family socio-economic status and children’s health and well-being outcomes.

### Narrative Synthesis

The findings of this review underscore the multifaceted ways in which neighborhoods shape child well-being through both direct and indirect pathways. The studies reviewed emphasize that positive neighborhood attributes, including safety, social cohesion, accessible play spaces, and supportive institutions, collectively foster environments conducive to children's flourishing. The studies included in this review employed a range of research methodologies, reflecting the interdisciplinary nature of the field. The methodological diversity of the included studies highlights the strengths of combining large-scale quantitative evidence with rich, contextual qualitative findings to provide a comprehensive understanding of how neighborhoods shape child well-being. Quantitative studies tend to focus on one aspect of neighborhood quality in relation to child well-being such as safety or spaces for play, specifically for testing hypotheses. The resources investigated in these studies tend to relate to collective efficacy or social cohesion. Qualitative studies, on the other hand, leave more room for explorative approaches, with children bringing a variety of topics to the forefront. In many qualitative papers we see that children include physical, social and institutional resources. They also place a strong emphasis on spaces facilitating social interaction with their peers as well as pointing to the importance of safety.

#### Social Resources for Well-Being

Social neighborhood characteristics, particularly social cohesion, supportive relationships, and perceptions of safety, are related to children's well-being. Studies in this review highlighted the importance of social support from non-parental adults, including teachers, neighbors, and community members, in fostering resilience and life satisfaction (Gadermann et al., 2016; Oberle et al., 2011). Moreover, neighborhood social cohesion, defined as trust, mutual support, and collective efficacy, was positively associated with children's emotional well-being, social adjustment, and flourishing (Barnhart et al., 2022; Kandasamy et al., 2018).

The role of peer relationships and community belonging on child well-being also emerged as critical. In particular, children who perceived strong connections with their peers within the neighborhood reported higher levels of subjective well-being and a sense of security (den Besten, 2010; Wang et al., 2023). Importantly, some studies found that neighborhood safety moderated these effects, with children in safer communities experiencing greater benefits from social cohesion and peer connections (Ditzel et al., 2023).

#### Physical Resources for Well-Being

Physical neighborhood characteristics, such as playgrounds, parks, sidewalks, and green spaces, emerged as essential for fostering positive child outcomes. Studies demonstrated that children who had access to safe and well-maintained outdoor spaces reported higher life satisfaction, social well-being, and engagement in physical activities. In particular, safe play areas were associated with greater opportunities for peer interactions, physical exercise, and emotional regulation (Alfaro et al., 2024; Corominas et al., 2021; Ditzel et al., 2023). Also, access to and quality of greenery are consistently associated with better child well-being. Green spaces support children’s well-being both directly and indirectly, through increased opportunities for outdoor play, physical activity, social interaction, and enhanced perceptions of safety and neighborhood satisfaction. Evidence also suggests that improvements in green spaces (e.g. park renovations) may yield stronger benefits, specifically for children from socio-economically disadvantaged backgrounds (Hong et al., 2025). This may highlight green space as a potential lever for reducing inequalities in child well-being. Additionally, neighborhood cleanliness and infrastructure quality (e.g., well-maintained sidewalks and public transport connectivity) contributed to children's perceptions of safety and comfort, enhancing their overall subjective well-being (Lee & Yoo, 2015; van den Berg et al., 2020).

#### Institutional Resources for Well-Being

Institutional factors, including access to educational, recreational, and community resources, were also found to significantly impact child well-being. Children in neighborhoods with libraries, youth clubs, sports facilities, and extracurricular activities had better psychological adjustment, higher academic engagement, and increased overall well-being (Ashiabi, 2018; Rogers, 2012; Ucci et al., 2022). Moreover, access to structured after-school programs and community centers was linked to positive social development and reduced stress among children (Shortt & Ross, 2021).

Beyond structured institutions, the review also highlighted the importance of informal spaces for social interaction, such as local gathering spots, convenience stores, and community hubs, in shaping children's neighborhood experiences (Horgan et al., 2023). These spaces played a pivotal role in fostering a sense of community and enabling spontaneous peer interactions, which, in turn, enhanced children's sense of belonging and emotional security.

### Interconnected Resources and Moderating Factors

A key finding across studies is that neighborhood characteristics do not operate in isolation but interact in complex ways to shape child well-being. Safety emerged as a critical cross-cutting factor, with perceived safety amplifying the positive effects of both social and physical resources. For example, neighborhoods with high social cohesion and well-maintained physical infrastructure provided a stronger buffer against adversity compared to those lacking these features (Wang et al., 2023; Witten et al., 2019).

Additionally, socioeconomic factors moderated the impact of neighborhood resources on child well-being. Children from lower-income neighborhoods often benefited the most from improved physical and social environments, as these factors helped counteract broader structural inequalities (González-Carrasco et al., 2019; Hong et al., 2025). However, disparities in access to resources such as parks, after-school programs, and safe public spaces highlighted ongoing inequities in neighborhood conditions (Eriksson & Dahlblom, 2020). Children themselves were also conscious about the importance of safety on their experience of the neighborhood as well as the disparities in access to neighborhood resources (Shortt & Ross, 2021).

## Discussion

This review contributes to a growing body of literature emphasizing the importance of neighborhoods for children’s well-being (Christian et al., 2015; Leventhal & Dupéré, 2019; Mayne et al., 2021). The review catalogues cross-disciplinary empirical evidence on which neighborhood characteristics influence child well-being, when and how. Our findings not only confirm the significance of different neighborhoods for child well-being and highlight the interconnectedness of resources but also provide a nuanced understanding of their mechanisms and emphasize the role of children’s agency in shaping these environments. Moving beyond the traditional deficit-focused perspective, our findings highlight that neighborhoods, with available and accessible resources, can serve as reservoirs of support for children's well-being.

In addition to its theoretical grounding in ecological and family systems theories, this review can be positioned within established, multidimensional child well-being measurement frameworks frequently used in child indicators research, including those proposed by Ben-Arieh (2000), UNICEF, and the OECD. These frameworks typically distinguish domains such as education, social relationships, safety, and health, and recognize that these domains are interconnected and interact with one another. The neighborhood characteristics and mechanisms identified in this review correspond to these domains while at the same time extending them by offering a process-oriented perspective on how child well-being outcomes are produced through interactions between neighborhood resources, family systems, and children’s agency. In this way, the review contributes not only to theoretical understanding but also to the conceptual grounding and interpretation of child well-being indicators.

Neighborhoods can foster child well-being by having spaces and places that facilitate play, exercise, talent development, and social interaction, such as play areas, green areas, youth clubs and sports facilities. Beyond the presence of such amenities, neighborhood infrastructure, such as safe walking and cycling routes, traffic-calming measures, adequate lighting and proximity to these amenities, plays a crucial role in enabling children and families to make use of these resources. This is particularly important as public and green spaces are increasingly under pressure due to densification and competing land uses in cities worldwide (Chitrakar et al., 2022; Husqvarna Group, 2024; Low, 2023). Climate change may further exacerbate this pressure, for example through heat stress, flooding or reduced usability of outdoor spaces (Orsetti et al., 2022). At the same time, children’s everyday lives have increasingly shifted indoors, accompanied by a decline in unsupervised outdoor play, physical activity, risk-taking behaviors, and contact with the natural environment (Chawla, 2020; Harper, 2017). Together, these developments highlight the importance of well-designed, climate-resilient neighborhood infrastructure that supports children’s independent mobility and sustained engagement with outdoor environments.

Moreover, neighborhoods that actively support families, via social cohesion, accessible activities, and informal and formal support networks, can promote well-being at multiple, interacting levels. In cohesive neighborhoods, families may be more inclined to engage with neighbors, exchange support and make use of local services, thereby strengthening both individual and collective resources. Such processes can generate positive feedback loops in which parental well-being, parenting practices and child well-being mutually reinforce one another, while simultaneously deepening social cohesion within the neighborhood. Conceptualizing families as systems embedded within broader neighborhood systems allows researchers to move beyond linear mediation models toward more dynamic, transactional frameworks that acknowledge bidirectional influences. Empirical evidence shows that residents’ mental health and social cohesion affect each other over time, with mental health often preceding (and potentially shaping) neighborhood cohesion rather than merely resulting from it (Prati, 2024). This underscores the need to examine neighborhood resources and child well-being as evolving, interdependent processes rather than static conditions. Family research further encourages a temporal perspective, emphasizing how families adapt to changing environmental contexts while neighborhoods themselves continuously transform. In line with this view, scholars have advocated for stronger integration of research, policy, and intervention design, particularly through context-specific neighborhood initiatives that incorporate social processes and organizational infrastructure (McDonell & Sianko, 2021). Promising examples include community-wide prevention programs such as *Strong Communities for Children* (Kimbrough-Melton & Campbell, 2008). This relates to one of the most interesting findings from our cross-disciplinary synthesis: the interconnectedness of neighborhood resources. Social cohesion enhances the positive impact of physical infrastructure; safe and well-maintained parks enable peer relationships; and access to supportive institutions strengthens the effects of community trust (den Besten, 2010; Horgan et al., 2023; Wang et al., 2023; Witten et al., 2019). Interestingly, there was not one single component that completely determined the behavior of the whole neighborhood system. For successful translation of insights about important neighborhood factors it is therefore important to embrace this complexity. For example, by adopting complexity science or assemblage thinking and by using an iterative approach, seeing change as an ongoing process (e.g., placemaking), rather than trying to design a fully formed and static plan (Istoriou, & Pozoukidou, 2024).

The studies included in this review also shed light on the mechanisms through which neighborhood resources affect children's well-being. Mediators like family resilience, lower parental stress, and out-of-school activities highlight the layered pathways through which neighborhood contexts exert influence. These mechanisms include for example, access to play areas promoting physical activity and peer interaction, and in turn well-being (Rogers, 2012), and neighborhood cohesion reducing maternal stress, which in turn supports child well-being (Fava et al., 2022). Many of the identified mediators, such as parenting stress (Ashiabi, 2018), parental well-being (Ashiabi, 2018; Fava et al., 2022), family processes (Ashiabi, 2018), and family resilience (Barnhart et al., 2022), are situated in the family system. These findings can be understood through the lens of family studies. Children are part of a family system (family systems theory; Cox & Paley, 1997), which in turn is embedded in the neighborhood (Bronfenbrenner, 2005). Integrating both frameworks underscores that neighborhood effects on children are not direct but rather filtered through family-level processes and feedback loops within the family system. This interplay invites more nuanced theorizing about how contextual adversity or support is processed within families and transmitted to children (Noah, 2015; Riina, 2024). From an indicator perspective, these findings underscore that neighborhood-level indicators should not be interpreted in isolation, but in relation to family-level mediators. By explicating these pathways, the present synthesis helps explain why similar neighborhood conditions may be associated with different child well-being outcomes across contexts, thereby informing both indicator selection and interpretation.

Moreover, and related, this review highlights the importance of a contextualized understanding of neighborhood factors and how they may affect child well-being. Country and cultural context were indicated as moderators of the relation between neighborhood factors and child outcomes, with some countries showing significant effects for certain resources and not for others (Ditzel et al., 2023; González-Carrasco et al., 2019; Lawler et al., 2018). Some insights and implications of these insights may not be transportable to other countries or even other communities within the same country. In translating research findings to policy and practice it is important to include knowledge about the specific target neighborhood or community (Powell et al., 2024) and by taking into the account the moderators that this review study have brought to light.

From a methodological perspective, the interdisciplinary nature of this review is one of its main strengths. Insights from sociology, health, pedagogy, psychology, and urban planning also enriched our understanding of child well-being as a multifaceted construct, encompassing subjective happiness, life satisfaction, health, social adjustment, and academic self-efficacy. These diverse disciplinary lenses revealed different emphasis, for instance, sociology’s focus on belonging and cohesion versus health's focus on functional quality of life. Engaging with these different paradigms also pointed to disciplinary blind spots, such as the relative under-theorization of space in psychology or the limited attention to lived experience in some health research, bringing these findings together can unravel some of the complexities at play when seeking to advance child well-being on the neighborhood level.

Importantly, this review reinforces the longstanding insight that children are not passive recipients of their environments, but active agents in shaping how they experience and respond to them. In studies in which children were involved as co-researchers (Shortt & Ross, 2021; Turner et al., 2006), children showed agency in the ways that they navigate their neighborhoods and use the resources available to them. In qualitative and participatory studies, children emphasized aspects of neighborhood life often overlooked by other research, including the importance of informal social spaces, feelings of safety, and opportunities for peer interaction (den Besten, 2010; Eriksson & Dahlblom, 2020; Horgan et al., 2023). This review shows that children’s voices can improve our understanding of the neighborhood as a resource and overall underlines the importance of involving children in the research on their living environments.

Finally, equity emerged as a cross-cutting theme. Children in socioeconomically disadvantaged areas often benefit the most from neighborhood resources but also face the greatest barriers to access those (González-Carrasco et al., 2019; Hong et al., 2025). Addressing these structural inequities is essential. Improvements in neighborhood environments should prioritize accessibility, affordability, and inclusivity. Especially since perceptions of exclusion or stigma can erode the benefits of otherwise well-resourced environments (Shortt & Ross, 2021). Last, children were found to be active users of neighborhood spaces as well as having profound insights into how neighborhood resources potentially impact them, their unique understanding of their lived experience holds the potential for useful and contextual input and should be included, for both researchers and policy makers.

This review focused on organizing, categorizing and descriptively integrating the literature, rather than evaluating effect sizes or establishing causal relationships. As a result, null findings, contradictory evidence, and unintended or adverse effects of neighborhood characteristics were not systematically synthesized. The findings should therefore not be interpreted as indicating that specific neighborhood resources are uniformly beneficial, nor that strengthening such resources will necessarily lead to positive outcomes across contexts. Instead, this review identifies neighborhood characteristics that have been empirically associated with positive child well-being outcomes under certain conditions, highlighting them as priorities for further investigation regarding when, for whom, and under which circumstances they may function as resources rather than risks or effective targets for prevention and intervention. While this approach aligns with an asset-based perspective, it may obscure the complexity of neighborhood effects. For instance, neighborhood green space has also been linked to higher levels of externalizing behavior in adolescence (Kotlaja et al., 2018; Weeland et al., 2019), possibly because greener areas may offer shielded spaces that facilitate unsupervised activities away from adult oversight. Future studies should therefore more explicitly examine the contextual conditions under which neighborhood characteristics operate as protective factors versus risk factors.

## Conclusions and Implications

From a policy and practice perspective, the findings of this review suggest that strengthening neighborhood resources requires attention not only to their presence, but also to how they are activated in everyday social life in a contextualized way. Research distinguishes between residents’ perceptions of their neighborhood (e.g., trust, safety, shared norms) and everyday practices of “neighborliness,” such as helping, greeting, organizing, or spending time with neighbors. While both contribute to neighborhood social capital as a broader social construct reflecting collective norms, sentiments, and beliefs, neighborliness can be understood as its behavioral expression (McDonell & Sianko, 2021). This distinction is important because interventions may target social relations at different levels: fostering positive perceptions alone may be insufficient if they do not translate into opportunities for social interaction and shared practices.

At the local level, this implies that child- and family-oriented neighborhood initiatives may benefit from small-scale, low-threshold interventions that enable residents to “do things together,” such as shared use of public space, neighborhood play initiatives, community events, or the co-design of playgrounds and green spaces with children and caregivers. Such initiatives do not necessarily require large infrastructural investments but can strengthen social cohesion by making neighborliness visible and practicable in everyday life.

At the municipal or national level, policies aimed at child well-being may consider embedding neighborhood-based social infrastructure into broader welfare, education, and urban planning strategies. This includes safeguarding access to informal meeting places, libraries, youth clubs, and green spaces, particularly in socioeconomically disadvantaged areas, and supporting professionals (e.g., community workers, teachers, youth workers) who act as connectors between families, institutions, and neighborhood networks.

At the international level, the findings underscore the relevance of context-sensitive, place-based approaches rather than one-size-fits-all solutions. While concepts such as social cohesion or neighborhood quality are widely used, their practical meaning varies across cultural, institutional, and spatial contexts. Comparative child well-being frameworks and indicator systems may therefore benefit from incorporating not only structural indicators (e.g., availability of facilities) but also indicators capturing children’s and families’ lived experiences of accessibility, safety, and everyday social interaction within their neighborhoods.

This review underscores that neighborhoods are not just passive settings, but dynamic, relational spaces that can support child development. Neighborhoods are complex systems, consisting of various interconnecting components (e.g., people, places and institutions) that interact in various ways (Grossmann & Haase, 2016). The interconnectedness of resources and relationships underscores the need for integrated neighborhood planning where interventions across domains are coordinated rather than isolated. By adopting a strength-based perspective and centering children’s voices, we can expand our understanding of how neighborhoods can become reservoirs of potential for well-being and resilience. These findings call for a paradigm shift: policies and interventions should not only aim to mitigate risk but also actively leverage neighborhood assets through integrated planning, participatory design, and contextual, equity-focused approaches.

## Supplementary Information

Below is the link to the electronic supplementary material.

Supplementary file1 (DOCX 116 KB)

Supplementary file2 (DOCX 120 KB)

## Data Availability

No datasets were generated or analysed during the current study.
