# Supplementary material for: The Neighborhood Resources that Shape Children’s Well-Being: A Systematic Literature Review
Source: Child Indic Res. 2026 Apr 13;19(4):1975–2013. doi: 10.1007/s12187-026-10362-x (PMC13570818; doi:10.1007/s12187-026-10362-x)
Supplement: Supplementary file 1 [file 12187_2026_10362_MOESM1_ESM.docx]

**Appendix Bias Checklist Quantitative Papers**

**Instrument**

We systematically evaluated the quality of the included quantitative studies by performing a bias check based on the Appraisal tool for Cross-Sectional Studies (AXIS; Downes et al., 2016). Since we also included longitudinal studies, we added one question regarding follow-up loss stemming from the National Institutes of Health Quality Assessment Tool for Observational Cohort and Cross-Sectional Studies (2014), namely:

21. Was loss to follow-up after baseline 20% or less?

*Explanation: Higher overall follow-up rates are considered better than lower follow-up rates, even though higher rates are expected in shorter studies, whereas lower overall follow-up rates are often seen in studies of longer duration. Usually, an acceptable overall follow-up rate is considered 80 percent or more of participants whose exposures were measured at baseline. However, this is just a general guideline. For example, a 6-month cohort study examining the relationship between dietary sodium intake and BP level may have over 90 percent follow-up, but a 20-year cohort study examining effects of sodium intake on stroke may have only a 65 percent follow-up rate.*

**Prompt ChatGPT (11 March 2025)**

*“I will now feed you a research article and an Appraisal tool for Cross-Sectional Studies, can you fill in the appraisal tool for this article, and (a) distinguish between yes, no, or no information available (is don't know in the tool) and (b) add comments describing on which information the yes, no, no information decision was based on. Please also take into account the description of the appraisal questions in the tool (p. 4 onwards) as an explanation of the questions. Please answer conservatively: if information about the question is not directly available or explicitly given then answer no information available an add comment. Add one extra question to the table, namely 21. Was loss to follow-up after baseline 20% or less? This is the explanation of this question: Higher overall followup rates are always better than lower follow-up rates, even though higher rates are expected in shorter studies, whereas lower overall follow-up rates are often seen in studies of longer duration. Usually, an acceptable overall follow-up rate is considered 80 percent or more of participants whose exposures were measured at baseline. However, this is just a general guideline. For example, a 6-month cohort study examining the relationship between dietary sodium intake and BP level may have over 90 percent follow-up, but a 20-year cohort study examining effects of sodium intake on stroke may have only a 65 percent follow-up rate. For this question the answer can also be not applicable in case the research was not longitudinal.*

*Add the answers to these questions to a table, a similar table to the one shown in the appraisal tool document. And conform the APA layout.*

*Name the table as the article reference according to APA 7 reference style.”*

**Table 1**

*Appraisal of Barnhart, Bode, Gearhart, and Maguire-Jack (2022) using the AXIS tool*

| **Question** | **Answer** | **Comment** |
| --- | --- | --- |
| **Introduction** |  |  |
| 1. Were the aims/objectives of the study clear? | Yes | The study clearly states its aim to examine how neighborhood assets promote child and adolescent flourishing through family resilience. |
| **Methods** |  |  |
| 2. Was the study design appropriate for the stated aim(s)? | Yes | A cross-sectional study design is appropriate for assessing associations between neighborhood factors, family resilience, and flourishing. |
| 3. Was the sample size justified? | No information available | The study does not explicitly provide a power calculation or justification for the sample size, though it uses a large dataset (NSCH). |
| 4. Was the target/reference population clearly defined? | Yes | The target population is clearly stated as U.S. children and adolescents aged 6–17. |
| 5. Was the sample frame taken from an appropriate population base so that it closely represented the target/reference population? | Yes | The sample was drawn from the National Survey of Children’s Health (NSCH), a representative dataset. |
| 6. Was the selection process likely to select subjects/participants that were representative of the target/reference population? | Yes | The use of NSCH ensures a representative selection process. |
| 7. Were measures undertaken to address and categorize non-responders? | No information available | The study does not provide details on non-responders or measures taken to account for their potential impact. |
| 8. Were the risk factor and outcome variables measured appropriate to the aims of the study? | Yes | Variables were relevant to assessing neighborhood influences, family resilience, and child/adolescent flourishing. |
| 9. Were the risk factor and outcome variables measured correctly using instruments/measurements that had been trialed, piloted, or published previously? | Yes | The study uses validated measures from the NSCH dataset, including standardized questions on flourishing, family resilience, and neighborhood cohesion. |
| 10. Is it clear what was used to determine statistical significance and/or precision estimates? | Yes | Statistical significance was assessed using p-values, confidence intervals, and structural equation modeling (SEM). |
| 11. Were the methods (including statistical methods) sufficiently described to enable them to be repeated? | Yes | The study provides a detailed description of SEM and other analytical approaches. |
| **Results** |  |  |
| 12. Were the basic data adequately described? | Yes | The study presents descriptive statistics and sample characteristics. |
| 13. Does the response rate raise concerns about non-response bias? | No information available | The response rate is not explicitly stated, making it unclear whether non-response bias is a concern. |
| 14. If appropriate, was information about non-responders described? | No | No details on non-responders were provided. |
| 15. Were the results internally consistent? | Yes | The results align with the theoretical framework and expected relationships between variables. |
| 16. Were the results presented for all the analyses described in the methods? | Yes | The study reports findings from all planned statistical analyses. |
| **Discussion** |  |  |
| 17. Were the authors' discussions and conclusions justified by the results? | Yes | The discussion is well-supported by the results, with interpretations consistent with findings. |
| 18. Were the limitations of the study discussed? | Yes | The study acknowledges limitations, including its cross-sectional design and potential unmeasured confounders. |
| **Other** |  |  |
| 19. Were there any funding sources or conflicts of interest that may affect the authors’ interpretation of the results? | No | The authors declare no conflicts of interest and state that the research received no external funding. |
| 20. Was ethical approval or consent of participants attained? | Yes | Ethical approval is not required for secondary data analysis, but the study acknowledges the ethical review process for NSCH data collection. |
| 21. Was loss to follow-up after baseline 20% or less? | Not applicable | The study is cross-sectional, so loss to follow-up is not relevant. |

**Table 2**

*Appraisal of Corominas, González-Carrasco, and Casas (2020) using the AXIS tool*

| **Question** | **Answer** | **Comment** |
| --- | --- | --- |
| **Introduction** |  |  |
| 1. Were the aims/objectives of the study clear? | Yes | The study clearly states its aim to identify factors ensuring an optimum play environment and analyze its relation to children’s subjective well-being. |
| **Methods** |  |  |
| 2. Was the study design appropriate for the stated aim(s)? | Yes | A cross-sectional design is suitable for assessing the relationship between play environments and subjective well-being. |
| 3. Was the sample size justified? | No information available | The study does not explicitly provide a justification or power calculation for the sample size, though it includes 3,962 children. |
| 4. Was the target/reference population clearly defined? | Yes | The target population is clearly defined as children aged 10-12 in Barcelona, Spain. |
| 5. Was the sample frame taken from an appropriate population base so that it closely represented the target/reference population? | Yes | The sample was drawn from a school-based probabilistic sample covering various districts in Barcelona. |
| 6. Was the selection process likely to select subjects/participants that were representative of the target/reference population? | Yes | The two-stage cluster sampling approach ensured representativeness of schoolchildren in Barcelona. |
| 7. Were measures undertaken to address and categorize non-responders? | No information available | The study does not provide details on non-responders or any measures taken to address them. |
| 8. Were the risk factor and outcome variables measured appropriate to the aims of the study? | Yes | Variables related to play environment, social well-being, and subjective well-being were appropriately selected based on existing frameworks. |
| 9. Were the risk factor and outcome variables measured correctly using instruments/measurements that had been trialed, piloted, or published previously? | Yes | The study uses validated indicators from the Children’s Worlds survey and other established scales. |
| 10. Is it clear what was used to determine statistical significance and/or precision estimates? | Yes | The study employs statistical tests such as cluster analysis, chi-square tests, and descriptive statistics, though no p-value thresholds are explicitly stated. |
| 11. Were the methods (including statistical methods) sufficiently described to enable them to be repeated? | Yes | The methodology, including data collection, sampling, and statistical techniques, is well-described. |
| **Results** |  |  |
| 12. Were the basic data adequately described? | Yes | The study provides detailed descriptive statistics of key indicators. |
| 13. Does the response rate raise concerns about non-response bias? | No information available | The response rate is not explicitly mentioned, making it unclear whether non-response bias is a concern. |
| 14. If appropriate, was information about non-responders described? | No | The study does not discuss non-responders or their characteristics. |
| 15. Were the results internally consistent? | Yes | The findings align with the theoretical framework and expected associations between play environment and well-being. |
| 16. Were the results presented for all the analyses described in the methods? | Yes | The study reports findings from all planned analyses, including cluster analysis and subgroup comparisons. |
| **Discussion** |  |  |
| 17. Were the authors' discussions and conclusions justified by the results? | Yes | The discussion is well-supported by the results, with conclusions grounded in the data. |
| 18. Were the limitations of the study discussed? | Yes | The study acknowledges limitations, including its cross-sectional nature and the absence of certain play environment indicators. |
| **Other** |  |  |
| 19. Were there any funding sources or conflicts of interest that may affect the authors’ interpretation of the results? | No | The authors declare no conflicts of interest and acknowledge institutional funding. |
| 20. Was ethical approval or consent of participants attained? | Yes | The study followed ethical guidelines, and participants were informed, though parental consent was not explicitly required. |
| 21. Was loss to follow-up after baseline 20% or less? | Not applicable | The study is cross-sectional, so loss to follow-up is not relevant. |

### **Table 3**

### *Appraisal of Lee and Yoo (2015) using the AXIS tool*

| **Question** | **Answer** | **Comment** |
| --- | --- | --- |
| 1. Were the aims/objectives of the study clear? | Yes | The study clearly states its aims: to examine family, school, and community factors related to children's subjective well-being and to compare these across nations. |
| 2. Was the study design appropriate for the stated aim(s)? | Yes | The cross-sectional design is appropriate for examining associations between environmental factors and subjective well-being across different countries. |
| 3. Was the sample size justified? | No | The study acknowledges limitations in generalizability due to convenience sampling but does not provide a clear justification for the sample size. |
| 4. Was the target/reference population clearly defined? | Yes | The study specifies that the target population is 12-year-old children from 11 countries. |
| 5. Was the sample frame taken from an appropriate population base so that it closely represented the target/reference population under investigation? | No | Most countries used convenience sampling rather than random sampling, limiting representativeness. |
| 6. Was the selection process likely to select subjects/participants that were representative of the target/reference population under investigation? | No | Only five countries used random sampling; the others relied on convenience sampling, which may introduce selection bias. |
| 7. Were measures undertaken to address and categorize non-responders? | No information available | The study does not discuss non-responders or how they were accounted for. |
| 8. Were the risk factor and outcome variables measured appropriately to the aims of the study? | Yes | The study used validated subjective well-being scales, including the General Domain Satisfaction Index (GDSI). |
| 9. Were the risk factor and outcome variables measured correctly using instruments/measurements that had been trialed, piloted, or published previously? | Yes | The GDSI has been used in multiple international studies. |
| 10. Is it clear what was used to determine statistical significance and/or precision estimates (e.g., p-values, confidence intervals)? | Yes | The study provides p-values and confidence intervals for regression analyses. |
| 11. Were the methods (including statistical methods) sufficiently described to enable them to be repeated? | Yes | The study describes the statistical methods used, including multiple regression and hierarchical linear modeling. |
| 12. Were the basic data adequately described? | Yes | The study provides descriptive statistics by country for key variables. |
| 13. Does the response rate raise concerns about non-response bias? | No information available | The study does not report response rates. |
| 14. If appropriate, was information about non-responders described? | No | The study does not mention non-responders. |
| 15. Were the results internally consistent? | Yes | The results align well with the study's aims and theoretical framework. |
| 16. Were the results presented for all the analyses described in the methods? | Yes | The study presents findings for all planned analyses. |
| 17. Were the authors' discussions and conclusions justified by the results? | Yes | The conclusions reflect the study's findings and acknowledge limitations. |
| 18. Were the limitations of the study discussed? | Yes | The authors discuss limitations related to sampling, generalizability, and measurement. |
| 19. Were there any funding sources or conflicts of interest that may affect the authors' interpretation of the results? | No information available | The study does not explicitly mention funding or conflicts of interest. |
| 20. Was ethical approval or consent of participants attained? | No information available | Ethical approval is not mentioned. |
| 21. Was loss to follow-up after baseline 20% or less? | Not applicable | This was a cross-sectional study, so follow-up loss is not relevant. |

**Table 4**

*Appraisal of Wu, Ohinmaa, and Veugelers (2010) using the AXIS Tool*

| **Question** |  | **Answer** | **Comments** |
| --- | --- | --- | --- |
| **Introduction** |  |  |  |
| 1. Were the aims/objectives of the study clear? |  | Yes | The study clearly aims to describe health-related quality of life (HRQOL) among grade-five students in Alberta, Canada, and examine sociodemographic and neighborhood determinants. |
| **Methods** |  |  |  |
| 2. Was the study design appropriate for the stated aim(s)? |  | Yes | A cross-sectional design is appropriate for assessing associations between HRQOL, sociodemographic factors, and neighborhood characteristics. |
| 3. Was the sample size justified? |  | No information available | The study does not explicitly justify the sample size, though it uses a large sample (n = 3,421). |
| 4. Was the target/reference population clearly defined? |  | Yes | The study specifies that it examines grade-five students in Alberta, Canada. |
| 5. Was the sample frame taken from an appropriate population base? |  | Yes | The study sample was drawn from a structured, population-based survey, ensuring representativeness. |
| 6. Was the selection process likely to select representative participants? |  | Yes | The study used stratified random sampling, which enhances representativeness. |
| 7. Were measures undertaken to address and categorize non-responders? |  | No information available | The study does not discuss non-responders explicitly. |
| 8. Were the risk factor and outcome variables measured appropriately? |  | Yes | The study uses the EQ-5D-Y, a validated measure of HRQOL. |
| 9. Were the risk factor and outcome variables measured using validated instruments? |  | Yes | The EQ-5D-Y is a well-established tool for measuring HRQOL. |
| 10. Is it clear what was used to determine statistical significance and/or precision estimates? |  | Yes | The study reports p-values and confidence intervals. |
| 11. Were the methods sufficiently described to enable replication? |  | Yes | The study describes the survey methodology and statistical analyses adequately. |
| **Results** |  |  |  |
| 12. Were the basic data adequately described? |  | Yes | The study provides descriptive statistics, including mean EQ-5D-Y scores. |
| 13. Does the response rate raise concerns about non-response bias? |  | No | The response rate is 61.2% of total grade-five student population in those schools. |
| 14. Was information about non-responders described? |  | No | No details about non-responders are provided. |
| 15. Were the results internally consistent? |  | Yes | Findings align with the study hypotheses and statistical analysis. |
| 16. Were the results presented for all the analyses described in the methods? |  | Yes | The study presents findings for all planned analyses. |
| **Discussion** |  |  |  |
| 17. Were the authors' discussions and conclusions justified by the results? |  | Yes | The conclusions align with the results and discuss implications. |
| 18. Were the limitations of the study discussed? |  | Yes | The study discusses potential limitations such as the cross-sectional design. |
| **Other** |  |  |  |
| 19. Were there any funding sources or conflicts of interest? |  | No | The study does not indicate conflicts of interest. |
| 20. Was ethical approval or consent of participants attained? |  | No information available | Ethical approval is not explicitly mentioned. |
| 21. Was loss to follow-up after baseline 20% or less? |  | Not applicable | The study is cross-sectional and does not involve follow-up. |

**Table 5**

*Appraisal of Zhou, Guo, and Lu (2020) using the AXIS Tool*

| **Question** | **Answer** | **Comments** |
| --- | --- | --- |
| **Introduction** |  |  |
| 1. Were the aims/objectives of the study clear? | Yes | The study examines the well-being of rural children in China as a function of parental absence, economic status, and neighborhood environment. |
| **Methods** |  |  |
| 2. Was the study design appropriate for the stated aim(s)? | Yes | A cross-sectional design is appropriate for identifying associations between these factors. |
| 3. Was the sample size justified? | No information available | The study does not explicitly justify its sample size, though it reports using 519 participants. |
| 4. Was the target/reference population clearly defined? | Yes | The study defines its population as rural children aged 5–9 in Henan Province, China. |
| 5. Was the sample frame appropriate? | Yes | Participants were selected from four rural primary schools, ensuring relevance to the research question. |
| 6. Was the selection process representative? | Yes | A structured approach to participant selection was used. |
| 7. Were non-responders categorized? | No information available | No details about non-responders are provided. |
| 8. Were variables measured appropriately? | Yes | The study employs validated instruments for assessing neighborhood cohesion and well-being. |
| 9. Were validated instruments used? | Yes | The study references established measures such as the neighborhood cohesion scale. |
| 10. Was statistical significance clearly reported? | Yes | The study provides p-values and confidence intervals. |
| 11. Were methods sufficiently described? | Yes | The study details its data collection process and statistical approach. |
| **Results** |  |  |
| 12. Were basic data adequately described? | Yes | The study presents descriptive statistics on key variables. |
| 13. Does response rate raise concerns about bias? | No information available | The response rate is not explicitly stated. |
| 14. Was information about non-responders described? | No | The study does not provide details on non-responders. |
| **Other** |  |  |
| 19. Were funding sources disclosed? | Yes | The study acknowledges funding from the Research Grants Council of Hong Kong. |
| 21. Was loss to follow-up after baseline 20% or less? | Yes | The study had a 3% attrition rate, indicating minimal follow-up loss. |

**Table 6**

*Appraisal of Witherspoon, Daniels, Mason, and Smith (2016) using the AXIS Tool*

| **Question** | **Answer** | **Comments** |
| --- | --- | --- |
| **Introduction** |  |  |
| 1. Were the aims/objectives of the study clear? | Yes | The study examines the relationship between neighborhood factors, racial-ethnic identity, and academic adjustment in children. |
| **Methods** |  |  |
| 2. Was the study design appropriate for the stated aim(s)? | Yes | A cross-sectional design is appropriate for identifying associations between neighborhood perceptions and racial-ethnic identity. |
| 3. Was the sample size justified? | No information available | The study does not explicitly justify its sample size, though it reports using 227 children. |
| 4. Was the target/reference population clearly defined? | Yes | The study defines its population as children in grades 2 through 5 attending afterschool programs. |
| 5. Was the sample frame appropriate? | Yes | The sample was drawn from diverse afterschool programs, ensuring representation. |
| 6. Was the selection process representative? | Yes | The study uses a structured approach to participant selection. |
| 7. Were non-responders categorized? | No information available | No details about non-responders are provided. |
| 8. Were variables measured appropriately? | Yes | The study employs validated instruments for assessing neighborhood perceptions and racial-ethnic identity. |
| 9. Were validated instruments used? | Yes | The study references previously validated measures of racial-ethnic identity and academic efficacy. |
| 10. Was statistical significance clearly reported? | Yes | The study provides p-values and confidence intervals. |
| 11. Were methods sufficiently described? | Yes | The study details its data collection process and statistical approach. |
| **Results** |  |  |
| 12. Were basic data adequately described? | Yes | The study presents descriptive statistics on key variables. |
| 13. Does response rate raise concerns about bias? | No | The response rate is 74% and this does not raise concerns about bias. |
| 14. Was information about non-responders described? | No | The study does not provide details on non-responders. |
| 15. Were the results internally consistent? | Yes | Findings align with the study hypotheses and statistical analysis. |
| 16. Were the results presented for all the analyses described in the methods? | Yes | The study presents findings for all planned analyses. |
| **Discussion** |  |  |
| 17. Were the authors' discussions and conclusions justified by the results? | Yes | The conclusions align with the results and discuss implications. |
| 18. Were the limitations of the study discussed? | Yes | The study discusses potential limitations such as the cross-sectional design. |
| **Other** |  |  |
| 19. Were there any funding sources or conflicts of interest? | No information available | The study does not explicitly mention funding or conflicts of interest. |
| 20. Was ethical approval or consent of participants attained? | No information available | Ethical approval is not explicitly mentioned. |
| 21. Was loss to follow-up after baseline 20% or less? | Not applicable | The study is cross-sectional and does not involve follow-up. |

**Table 7**

*Appraisal of Oberle, Schonert-Reichl, and Zumbo (2011) using the AXIS Tool*

| **Question** | **Answer** | **Comments** |
| --- | --- | --- |
| **Introduction** |  |  |
| 1. Were the aims/objectives of the study clear? | Yes | The study aims to examine how personal and ecological assets influence life satisfaction in early adolescence. |
| **Methods** |  |  |
| 2. Was the study design appropriate for the stated aim(s)? | Yes | A cross-sectional design is appropriate for assessing relationships between life satisfaction, optimism, and social support. |
| 3. Was the sample size justified? | No information available | The study does not explicitly justify the sample size, though it includes 1,402 participants. |
| 4. Was the target/reference population clearly defined? | Yes | The study focuses on 4th to 7th-grade students in Canada. |
| 5. Was the sample frame appropriate? | Yes | The study used stratified random sampling from multiple schools. |
| 6. Was the selection process representative? | Yes | Schools were selected based on neighborhood vulnerability levels to ensure diversity. |
| 7. Were non-responders categorized? | No information available | No discussion of non-responders is provided. |
| 8. Were variables measured appropriately? | Yes | The study uses validated measures, including the Satisfaction With Life Scale for Children (SWLS-C). |
| 9. Were validated instruments used? | Yes | The SWLS-C and other measures used in the study have been previously validated. |
| 10. Was statistical significance clearly reported? | Yes | The study provides p-values and model fit indices. |
| 11. Were methods sufficiently described? | Yes | Multilevel modeling (MLM) and statistical procedures are well detailed. |
| **Results** |  |  |
| 12. Were basic data adequately described? | Yes | The study presents descriptive statistics for key variables. |
| 13. Does response rate raise concerns about bias? | No | The response rate is 87% and this does not raise concerns about bias. |
| 14. Was information about non-responders described? | No | The study does not provide details on non-responders. |
| 15. Were the results internally consistent? | Yes | The findings align with theoretical frameworks and prior research. |
| 16. Were the results presented for all the analyses described in the methods? | Yes | The study reports all planned analyses and outcomes. |
| **Discussion** |  |  |
| 17. Were the authors' discussions and conclusions justified by the results? | Yes | The conclusions are supported by the reported findings. |
| 18. Were the limitations of the study discussed? | Yes | The study acknowledges its cross-sectional design as a limitation. |
| **Other** |  |  |
| 19. Were there any funding sources or conflicts of interest? | No information available | The study does not explicitly mention funding or conflicts of interest. |
| 20. Was ethical approval or consent of participants attained? | No information available | Ethical approval is not explicitly mentioned. |
| 21. Was loss to follow-up after baseline 20% or less? | Not applicable | The study is cross-sectional and does not involve follow-up. |

**Table 8**

*Appraisal of Oberle, Schonert-Reichl, Guhn, Zumbo, and Hertzman (2014) using the AXIS Tool*

| **Question** | **Answer** | **Comments** |
| --- | --- | --- |
| **Introduction** |  |  |
| 1. Were the aims/objectives of the study clear? | Yes | The study examines the role of supportive adults in emotional well-being among Grade 4 students in Vancouver. |
| **Methods** |  |  |
| 2. Was the study design appropriate for the stated aim(s)? | Yes | A cross-sectional design is appropriate for assessing associations between support factors and well-being. |
| 3. Was the sample size justified? | No information available | The study does not explicitly justify the sample size, though it includes 3,026 participants. |
| 4. Was the target/reference population clearly defined? | Yes | The study defines the population as Grade 4 students in Vancouver public schools. |
| 5. Was the sample frame appropriate? | Yes | The study includes students from 72 public schools, ensuring representativeness. |
| 6. Was the selection process representative? | Yes | The study uses a population-based sample from a large urban district. |
| 7. Were non-responders categorized? | No information available | No details about non-responders are provided. |
| 8. Were variables measured appropriately? | Yes | The study uses validated self-report measures of emotional well-being and adult support. |
| 9. Were validated instruments used? | Yes | The study references established psychological scales. |
| 10. Was statistical significance clearly reported? | Yes | The study provides p-values and statistical models. |
| 11. Were methods sufficiently described? | Yes | The study details its survey methodology and statistical approach. |
| **Results** |  |  |
| 12. Were basic data adequately described? | Yes | The study presents descriptive statistics for key variables. |
| 13. Does response rate raise concerns about bias? | No | The response rate is 93% and this does not raise concerns about bias. |
| 14. Was information about non-responders described? | No | The study does not provide details on non-responders. |
| 15. Were the results internally consistent? | Yes | The findings align with theoretical expectations and prior research. |
| 16. Were the results presented for all the analyses described in the methods? | Yes | The study reports all planned analyses and outcomes. |
| **Discussion** |  |  |
| 17. Were the authors' discussions and conclusions justified by the results? | Yes | The conclusions are supported by the findings. |
| 18. Were the limitations of the study discussed? | Yes | The study acknowledges its cross-sectional design and self-report limitations. |
| **Other** |  |  |
| 19. Were there any funding sources or conflicts of interest? | No information available | The study does not explicitly mention funding or conflicts of interest. |
| 20. Was ethical approval or consent of participants attained? | No information available | Ethical approval is not explicitly mentioned. |
| 21. Was loss to follow-up after baseline 20% or less? | Not applicable | The study is cross-sectional and does not involve follow-up. |

**Table 9**

*Appraisal of Fava, Panisch, Burke, Li, Spadola, and O’Driscoll (2022) using the AXIS Tool*

| **Question** | **Answer** | **Comments** |
| --- | --- | --- |
| **Introduction** |  |  |
| 1. Were the aims/objectives of the study clear? | Yes | The study examines the mediating effect of maternal well-being on the relationship between neighborhood perception and child well-being. |
| **Methods** |  |  |
| 2. Was the study design appropriate for the stated aim(s)? | Yes | A longitudinal design is appropriate for assessing mediation effects over time. |
| 3. Was the sample size justified? | No information available | The study does not explicitly justify the sample size, though it uses data from the Fragile Families and Child Wellbeing Study. |
| 4. Was the target/reference population clearly defined? | Yes | The study defines the population as children and mothers from the Fragile Families and Child Wellbeing Study. |
| 5. Was the sample frame appropriate? | Yes | The study uses a well-established longitudinal dataset. |
| 6. Was the selection process representative? | Yes | The Fragile Families dataset is designed to be representative of urban births in the U.S. |
| 7. Were non-responders categorized? | No information available | No details about non-responders are provided. |
| 8. Were variables measured appropriately? | Yes | The study uses validated measures of maternal and child well-being. |
| 9. Were validated instruments used? | Yes | The study references established psychological and social science scales. |
| 10. Was statistical significance clearly reported? | Yes | The study provides p-values and model fit indices. |
| 11. Were methods sufficiently described? | Yes | The study details its structural equation modeling approach. |
| **Results** |  |  |
| 12. Were basic data adequately described? | Yes | The study presents descriptive statistics for key variables. |
| 13. Does response rate raise concerns about bias? | No information available | The response rate is not explicitly stated. |
| 14. Was information about non-responders described? | No | The study does not provide details on non-responders. |
| 15. Were the results internally consistent? | Yes | The findings align with theoretical expectations and prior research. |
| 16. Were the results presented for all the analyses described in the methods? | Yes | The study reports all planned analyses and outcomes. |
| **Discussion** |  |  |
| 17. Were the authors' discussions and conclusions justified by the results? | Yes | The conclusions are supported by the findings. |
| 18. Were the limitations of the study discussed? | Yes | The study acknowledges its limitations, including self-report bias. |
| **Other** |  |  |
| 19. Were there any funding sources or conflicts of interest? | No information available | The study does not explicitly mention funding or conflicts of interest. |
| 20. Was ethical approval or consent of participants attained? | No | Approval from an Institutional Review Board was not required, as publicly available data were used. |
| 21. Was loss to follow-up after baseline 20% or less? | Yes | The study follows participants over multiple years and reports acceptable follow-up rates. |

**Table 10**

*Appraisal of Vanden Berg, Waygood, van de Craats, and Kemperman (2020) using the AXIS Tool*

| **Question** | **Answer** | **Comments** |
| --- | --- | --- |
| **Introduction** |  |  |
| 1. Were the aims/objectives of the study clear? | Yes | The study examines the factors influencing parental safety perception, satisfaction with school travel, and mood in primary school children in the Netherlands. |
| **Methods** |  |  |
| 2. Was the study design appropriate for the stated aim(s)? | Yes | A cross-sectional design is appropriate for assessing relationships between parental safety perception, travel satisfaction, and mood. |
| 3. Was the sample size justified? | No information available | The study does not explicitly justify the sample size, though it includes 660 children. |
| 4. Was the target/reference population clearly defined? | Yes | The study focuses on primary school children (ages 7–12) and their parents in the Netherlands. |
| 5. Was the sample frame appropriate? | Yes | The study uses data from 15 primary schools in Arnhem, ensuring diverse representation. |
| 6. Was the selection process representative? | Yes | The study selected schools from various urban density levels, improving representativeness. |
| 7. Were non-responders categorized? | No information available | No discussion of non-responders is provided. |
| 8. Were variables measured appropriately? | Yes | The study employs validated measures such as the Satisfaction with Travel Scale for Children (STS-C). |
| 9. Were validated instruments used? | Yes | The study references established measures for social cohesion, safety perception, and satisfaction. |
| 10. Was statistical significance clearly reported? | Yes | The study provides p-values, model fit indices, and path analysis results. |
| 11. Were methods sufficiently described? | Yes | The study describes its survey methodology, path analysis, and statistical techniques in detail. |
| **Results** |  |  |
| 12. Were basic data adequately described? | Yes | The study presents detailed descriptive statistics on key variables. |
| 13. Does response rate raise concerns about bias? | No information available | The study reports a 46% response rate but does not discuss potential bias. |
| 14. Was information about non-responders described? | No | The study does not provide details on non-responders. |
| 15. Were the results internally consistent? | Yes | The findings align with theoretical expectations and prior literature. |
| 16. Were the results presented for all the analyses described in the methods? | Yes | The study presents results for all planned analyses, including mediation effects. |
| **Discussion** |  |  |
| 17. Were the authors' discussions and conclusions justified by the results? | Yes | The conclusions are supported by the study’s findings and implications for policy. |
| 18. Were the limitations of the study discussed? | Yes | The study acknowledges limitations such as self-report bias and seasonal data collection. |
| **Other** |  |  |
| 19. Were there any funding sources or conflicts of interest? | No | The study explicitly states that no specific funding was received. |
| 20. Was ethical approval or consent of participants attained? | Yes | The study states that all parents provided informed consent before participation. |
| 21. Was loss to follow-up after baseline 20% or less? | Not applicable | The study is cross-sectional and does not involve follow-up. |

**Table 11**

*Appraisal of Newland, Lawler, Giger, Roh, and Carr (2014) using the AXIS Tool*

| **Question** | **Answer** | **Comments** |
| --- | --- | --- |
| **Introduction** |  |  |
| 1. Were the aims/objectives of the study clear? | Yes | The study examines predictors of children's subjective well-being in rural communities of the United States. |
| **Methods** |  |  |
| 2. Was the study design appropriate for the stated aim(s)? | Yes | A cross-sectional design is appropriate for assessing associations between contextual factors and well-being. |
| 3. Was the sample size justified? | No information available | The study does not explicitly justify the sample size, though it includes 1,286 children. |
| 4. Was the target/reference population clearly defined? | Yes | The study focuses on 5th and 7th-grade students in rural Midwestern communities. |
| 5. Was the sample frame appropriate? | Yes | The study uses a convenience sample from six rural school districts. |
| 6. Was the selection process representative? | No information available | The study does not specify whether the selection process ensured representativeness. |
| 7. Were non-responders categorized? | No information available | No discussion of non-responders is provided. |
| 8. Were variables measured appropriately? | Yes | The study employs validated measures, including an adapted version of the Children's Worlds survey. |
| 9. Were validated instruments used? | Yes | The Children's Worlds survey has been extensively tested for reliability and validity. |
| 10. Was statistical significance clearly reported? | Yes | The study provides p-values, regression models, and confidence intervals. |
| 11. Were methods sufficiently described? | Yes | The study details its survey methodology, measures, and statistical analysis techniques. |
| **Results** |  |  |
| 12. Were basic data adequately described? | Yes | The study presents detailed descriptive statistics for key variables. |
| 13. Does response rate raise concerns about bias? | No | The study reports a 99% response rate, reducing concerns about bias. |
| 14. Was information about non-responders described? | No | The study does not provide details on non-responders. |
| 15. Were the results internally consistent? | Yes | The findings align with theoretical expectations and prior research. |
| 16. Were the results presented for all the analyses described in the methods? | Yes | The study presents findings for all planned analyses, including regression models. |
| **Discussion** |  |  |
| 17. Were the authors' discussions and conclusions justified by the results? | Yes | The conclusions are supported by the findings and discuss implications for rural child well-being. |
| 18. Were the limitations of the study discussed? | Yes | The study acknowledges limitations such as the cross-sectional design and reliance on self-report data. |
| **Other** |  |  |
| 19. Were there any funding sources or conflicts of interest? | No information available | The study does not explicitly mention funding or conflicts of interest. |
| 20. Was ethical approval or consent of participants attained? | Yes | The study reports obtaining institutional review board (IRB) approval and parental consent. |
| 21. Was loss to follow-up after baseline 20% or less? | Not applicable | The study is cross-sectional and does not involve follow-up. |

**Table 12**

*Appraisal of Mínguez (2020) using the AXIS Tool*

| **Question** | **Answer** | **Comments** |
| --- | --- | --- |
| **Introduction** |  |  |
| 1. Were the aims/objectives of the study clear? | Yes | The study explores how contextual factors such as family, friends, school, and neighborhood relate to children's subjective well-being across four European countries. |
| **Methods** |  |  |
| 2. Was the study design appropriate for the stated aim(s)? | Yes | A cross-sectional design is appropriate for identifying associations between social relationships and well-being. |
| 3. Was the sample size justified? | No information available | The study does not explicitly justify its sample size, though it reports using 4,812 participants. |
| 4. Was the target/reference population clearly defined? | Yes | The study defines its population as children aged 9–12 from Spain, Germany, the United Kingdom, and Norway. |
| 5. Was the sample frame appropriate? | Yes | The study uses data from the International Survey of Children’s Well-Being (ISCWeB), a widely used dataset. |
| 6. Was the selection process representative? | No information available | The study does not clarify if the sampling strategy ensures full representativeness. |
| 7. Were non-responders categorized? | No information available | No discussion of non-responders is provided. |
| 8. Were variables measured appropriately? | Yes | The study uses validated instruments, including Russell’s core affect scale for subjective well-being. |
| 9. Were validated instruments used? | Yes | The study references established scales for measuring subjective well-being and social relationships. |
| 10. Was statistical significance clearly reported? | Yes | The study provides p-values and regression analysis results. |
| 11. Were methods sufficiently described? | Yes | The study details its survey methodology, statistical techniques, and regression models. |
| **Results** |  |  |
| 12. Were basic data adequately described? | Yes | The study presents descriptive statistics on subjective well-being and its correlates. |
| 13. Does response rate raise concerns about bias? | No information available | The response rate is not explicitly stated. |
| 14. Was information about non-responders described? | No | The study does not provide details on non-responders. |
| 15. Were the results internally consistent? | Yes | The findings align with theoretical frameworks and prior research. |
| 16. Were the results presented for all the analyses described in the methods? | Yes | The study reports all planned analyses, including cross-country comparisons. |
| **Discussion** |  |  |
| 17. Were the authors' discussions and conclusions justified by the results? | Yes | The conclusions are supported by the findings and discuss implications for policy. |
| 18. Were the limitations of the study discussed? | Yes | The study acknowledges its cross-sectional design and potential measurement limitations. |
| **Other** |  |  |
| 19. Were there any funding sources or conflicts of interest? | Yes | The study acknowledges funding from the Spanish Ministry of Science, Innovation, and Universities. |
| 20. Was ethical approval or consent of participants attained? | No information available | Ethical approval is not explicitly mentioned. |
| 21. Was loss to follow-up after baseline 20% or less? | Not applicable | The study is cross-sectional and does not involve follow-up. |

**Table 13**

*Appraisal of Wang, Cheng, Jiang, and Zhou (2023) using the AXIS Tool*

| **Question** | **Answer** | **Comments** |
| --- | --- | --- |
| **Introduction** |  |  |
| 1. Were the aims/objectives of the study clear? | Yes | The study examines the effects of neighborhood quality on children's subjective well-being, with out-of-school activities as a mediator and friendship quality as a moderator. |
| **Methods** |  |  |
| 2. Was the study design appropriate for the stated aim(s)? | Yes | A cross-sectional design is appropriate for assessing associations between neighborhood quality, activities, and well-being. |
| 3. Was the sample size justified? | No information available | The study does not explicitly justify its sample size, though it includes 93,344 children. |
| 4. Was the target/reference population clearly defined? | Yes | The study focuses on children from 35 countries who participated in the third wave of the International Survey of Children’s Well-Being (ISCWeB). |
| 5. Was the sample frame appropriate? | Yes | The study uses a large international dataset, ensuring broad representation. |
| 6. Was the selection process representative? | No information available | The study does not specify if the sampling method ensures full representativeness across countries. |
| 7. Were non-responders categorized? | No information available | No discussion of non-responders is provided. |
| 8. Were variables measured appropriately? | Yes | The study employs validated instruments for neighborhood quality, friendship quality, and subjective well-being. |
| 9. Were validated instruments used? | Yes | The study references established scales for assessing subjective well-being and social relationships. |
| 10. Was statistical significance clearly reported? | Yes | The study provides p-values, regression models, and confidence intervals. |
| 11. Were methods sufficiently described? | Yes | The study details its survey methodology, mediation analysis, and moderation models. |
| **Results** |  |  |
| 12. Were basic data adequately described? | Yes | The study presents descriptive statistics and correlation tables for key variables. |
| 13. Does response rate raise concerns about bias? | No information available | The response rate is not explicitly stated. |
| 14. Was information about non-responders described? | No | The study does not provide details on non-responders. |
| 15. Were the results internally consistent? | Yes | The findings align with theoretical expectations and prior literature. |
| 16. Were the results presented for all the analyses described in the methods? | Yes | The study presents findings for all planned analyses, including mediation and moderation effects. |
| **Discussion** |  |  |
| 17. Were the authors' discussions and conclusions justified by the results? | Yes | The conclusions are supported by the findings and discuss implications for policy and interventions. |
| 18. Were the limitations of the study discussed? | Yes | The study acknowledges its cross-sectional design and reliance on self-reported data. |
| **Other** |  |  |
| 19. Were there any funding sources or conflicts of interest? | Yes | The study reports funding from the Chinese Ministry of Education. |
| 20. Was ethical approval or consent of participants attained? | Yes | The study states that ethical approval was obtained and informed consent was secured from participants. |
| 21. Was loss to follow-up after baseline 20% or less? | Not applicable | The study is cross-sectional and does not involve follow-up. |

**Table 14**

*Appraisal of Liu, Kia-Keating, Santacrose, and Modir (2023) using the AXIS Tool*

| **Question** | **Answer** | **Comments** |
| --- | --- | --- |
| **Introduction** |  |  |
| 1. Were the aims/objectives of the study clear? | Yes | The study explores associations between neighborhood elements and children's health and well-being in a nationally representative U.S. sample. |
| **Methods** |  |  |
| 2. Was the study design appropriate for the stated aim(s)? | Yes | A cross-sectional design is appropriate for identifying associations between neighborhood characteristics and child well-being. |
| 3. Was the sample size justified? | No information available | The study does not explicitly justify its sample size, though it analyzes data from a large sample (N = 49,513,974, ages 6–17). |
| 4. Was the target/reference population clearly defined? | Yes | The study defines its population as U.S. children aged 6–17 from the 2011–2012 National Survey of Children’s Health (NSCH). |
| 5. Was the sample frame appropriate? | Yes | The study uses data from the NSCH, a well-established national survey. |
| 6. Was the selection process representative? | Yes | The study uses weighted data to adjust for probability of selection and nonresponse bias. |
| 7. Were non-responders categorized? | No information available | No discussion of non-responders is provided. |
| 8. Were variables measured appropriately? | Yes | The study employs validated measures of neighborhood cohesion, health, and flourishing. |
| 9. Were validated instruments used? | Yes | The study references established measures such as those used in prior NSCH-based research. |
| 10. Was statistical significance clearly reported? | Yes | The study provides p-values, confidence intervals, and latent class analysis (LCA) model fit indices. |
| 11. Were methods sufficiently described? | Yes | The study details its LCA approach, covariate adjustments, and statistical techniques. |
| **Results** |  |  |
| 12. Were basic data adequately described? | Yes | The study presents descriptive statistics, model fit indices, and class distributions. |
| 13. Does response rate raise concerns about bias? | No information available | The study does not explicitly state the response rate. |
| 14. Was information about non-responders described? | No | The study does not provide details on non-responders. |
| 15. Were the results internally consistent? | Yes | The findings align with theoretical frameworks and prior research on neighborhood effects. |
| 16. Were the results presented for all the analyses described in the methods? | Yes | The study reports all planned analyses, including LCA-derived neighborhood profiles. |
| **Discussion** |  |  |
| 17. Were the authors' discussions and conclusions justified by the results? | Yes | The conclusions are supported by the findings and discuss implications for interventions. |
| 18. Were the limitations of the study discussed? | Yes | The study acknowledges its cross-sectional design and reliance on parent-reported data. |
| **Other** |  |  |
| 19. Were there any funding sources or conflicts of interest? | No information available | The study does not explicitly mention funding or conflicts of interest. |
| 20. Was ethical approval or consent of participants attained? | No information available | Ethical approval is not explicitly mentioned. |
| 21. Was loss to follow-up after baseline 20% or less? | Not applicable | The study is cross-sectional and does not involve follow-up. |

**Table 15**

*Appraisal of Lawler, Choi, Yoo, Lee, Roh, Newland, Giger, Sudhagoni, Brockevelt, and Lee (2018) using the AXIS Tool*

| **Question** | **Answer** | **Comments** |
| --- | --- | --- |
| **Introduction** |  |  |
| 1. Were the aims/objectives of the study clear? | Yes | The study examines subjective well-being in children from rural South Korea and the United States, using an ecological, relationship-based model. |
| **Methods** |  |  |
| 2. Was the study design appropriate for the stated aim(s)? | Yes | A cross-sectional design is appropriate for identifying relationships between individual and contextual factors and well-being. |
| 3. Was the sample size justified? | No information available | The study does not explicitly justify its sample size, though it includes 489 children from South Korea and 1,286 from the United States. |
| 4. Was the target/reference population clearly defined? | Yes | The study defines its population as children aged 10–12 in rural South Korea and the rural United States. |
| 5. Was the sample frame appropriate? | Yes | The study utilizes the Children's Worlds Survey, a well-established dataset. |
| 6. Was the selection process representative? | No information available | The study does not specify how schools were selected beyond broad rural classifications. |
| 7. Were non-responders categorized? | No information available | No discussion of non-responders is provided. |
| 8. Were variables measured appropriately? | Yes | The study employs validated instruments for family relationships, parent involvement, neighborhood quality, school satisfaction, and subjective well-being. |
| 9. Were validated instruments used? | Yes | The study references previously validated scales, including the Children's Worlds survey measures. |
| 10. Was statistical significance clearly reported? | Yes | The study provides p-values, structural equation modeling (SEM) fit indices, and path coefficients. |
| 11. Were methods sufficiently described? | Yes | The study details its survey methodology, SEM approach, and model specification. |
| **Results** |  |  |
| 12. Were basic data adequately described? | Yes | The study presents descriptive statistics for key variables in each country. |
| 13. Does response rate raise concerns about bias? | No information available | The study does not explicitly state response rates. |
| 14. Was information about non-responders described? | No | The study does not provide details on non-responders. |
| 15. Were the results internally consistent? | Yes | The findings align with theoretical models and prior research. |
| 16. Were the results presented for all the analyses described in the methods? | Yes | The study presents findings for all planned analyses, including SEM models. |
| **Discussion** |  |  |
| 17. Were the authors' discussions and conclusions justified by the results? | Yes | The conclusions are supported by the findings and discuss cultural implications. |
| 18. Were the limitations of the study discussed? | Yes | The study acknowledges limitations, including the cross-sectional design and self-report measures. |
| **Other** |  |  |
| 19. Were there any funding sources or conflicts of interest? | No | The study explicitly states that there are no conflicts of interest. |
| 20. Was ethical approval or consent of participants attained? | Yes | The study states that ethical approval was obtained, and informed consent was collected from participants and parents. |
| 21. Was loss to follow-up after baseline 20% or less? | Not applicable | The study is cross-sectional and does not involve follow-up. |

**Table 16**

*Appraisal of Gadermann, Guhn, Schonert-Reichl, Hymel, Thomson, and Hertzman (2016) using the AXIS Tool*

| **Question** | **Answer** | **Comments** |
| --- | --- | --- |
| **Introduction** |  |  |
| 1. Were the aims/objectives of the study clear? | Yes | The study investigates the influence of social relationships, health-related activities, and income on children's well-being and health. |
| **Methods** |  |  |
| 2. Was the study design appropriate for the stated aim(s)? | Yes | A cross-sectional design is appropriate for examining associations between well-being, health behaviors, and socioeconomic factors. |
| 3. Was the sample size justified? | No information available | The study does not explicitly justify its sample size, though it includes 5,026 students. |
| 4. Was the target/reference population clearly defined? | Yes | The study focuses on Grade 4 students in Canada. |
| 5. Was the sample frame appropriate? | Yes | The study used a population-based survey across three diverse school districts. |
| 6. Was the selection process representative? | Yes | Schools were selected to ensure demographic diversity. |
| 7. Were non-responders categorized? | No information available | No discussion of non-responders is provided. |
| 8. Were variables measured appropriately? | Yes | The study uses validated measures, including the Middle Years Development Instrument (MDI). |
| 9. Were validated instruments used? | Yes | The MDI and other scales have established reliability and validity. |
| 10. Was statistical significance clearly reported? | Yes | The study provides p-values and regression analysis results. |
| 11. Were methods sufficiently described? | Yes | Statistical analyses and mediation models are well-detailed. |
| **Results** |  |  |
| 12. Were basic data adequately described? | Yes | The study presents detailed descriptive statistics. |
| 13. Does response rate raise concerns about bias? | No | The response rates were 93%, 88%, and 99% across schools; this does not raise concerns about bias. |
| 14. Was information about non-responders described? | No | The study does not provide details on non-responders. |
| 15. Were the results internally consistent? | Yes | Findings align with theoretical expectations and previous research. |
| 16. Were the results presented for all the analyses described in the methods? | Yes | All planned analyses are reported, including mediation models. |
| **Discussion** |  |  |
| 17. Were the authors' discussions and conclusions justified by the results? | Yes | The conclusions are supported by the findings. |
| 18. Were the limitations of the study discussed? | Yes | The study acknowledges limitations such as self-report bias. |
| **Other** |  |  |
| 19. Were there any funding sources or conflicts of interest? | No information available | The study does not explicitly mention funding or conflicts of interest. |
| 20. Was ethical approval or consent of participants attained? | Yes | The study states that informed consent was obtained from parents. |
| 21. Was loss to follow-up after baseline 20% or less? | Not applicable | The study is cross-sectional and does not involve follow-up. |

**Table 17**

*Appraisal of González-Carrasco, Casas, Ben-Arieh, Savahl, and Tiliouine (2019) using the AXIS Tool*

| **Question** | **Answer** | **Comments** |
| --- | --- | --- |
| **Introduction** |  |  |
| 1. Were the aims/objectives of the study clear? | Yes | The study examines changes in children's subjective well-being across different age groups using data from the Children’s Worlds Survey. |
| **Methods** |  |  |
| 2. Was the study design appropriate for the stated aim(s)? | Yes | A cross-sectional design is appropriate for comparing well-being across different age groups. |
| 3. Was the sample size justified? | No information available | The study does not explicitly justify the sample size, though it includes data from multiple countries. |
| 4. Was the target/reference population clearly defined? | Yes | The study focuses on children aged 8, 10, and 12 from multiple countries participating in the Children’s Worlds Survey. |
| 5. Was the sample frame appropriate? | Yes | The study uses a large, well-established international dataset. |
| 6. Was the selection process representative? | No information available | The study does not specify how schools or children were selected within countries. |
| 7. Were non-responders categorized? | No information available | No discussion of non-responders is provided. |
| 8. Were variables measured appropriately? | Yes | The study employs validated measures of subjective well-being, satisfaction, and psychological factors. |
| 9. Were validated instruments used? | Yes | The study references established scales from the Children’s Worlds project. |
| 10. Was statistical significance clearly reported? | Yes | The study provides p-values and comparative statistics. |
| 11. Were methods sufficiently described? | Yes | The study details its statistical approach and comparative framework. |
| **Results** |  |  |
| 12. Were basic data adequately described? | Yes | The study presents descriptive statistics and cross-country comparisons. |
| 13. Does response rate raise concerns about bias? | No information available | The study does not report response rates. |
| 14. Was information about non-responders described? | No | The study does not provide details on non-responders. |
| 15. Were the results internally consistent? | Yes | Findings align with theoretical expectations and previous research. |
| 16. Were the results presented for all the analyses described in the methods? | Yes | The study presents findings for all planned analyses. |
| **Discussion** |  |  |
| 17. Were the authors' discussions and conclusions justified by the results? | Yes | The conclusions are supported by the findings. |
| 18. Were the limitations of the study discussed? | Yes | The study acknowledges limitations such as cultural differences in well-being assessments. |
| **Other** |  |  |
| 19. Were there any funding sources or conflicts of interest? | No information available | The study does not explicitly mention funding or conflicts of interest. |
| 20. Was ethical approval or consent of participants attained? | No information available | Ethical approval is not explicitly mentioned. |
| 21. Was loss to follow-up after baseline 20% or less? | Not applicable | The study is cross-sectional and does not involve follow-up. |

**Table 18**

*Appraisal of Kandasamy, Hirai, Ghandour, and Kogan (2018) using the AXIS Tool*

| **Question** | **Answer** | **Comments** |
| --- | --- | --- |
| **Introduction** |  |  |
| 1. Were the aims/objectives of the study clear? | Yes | The study examines factors associated with parental perception of flourishing in school-aged children in the U.S. |
| **Methods** |  |  |
| 2. Was the study design appropriate for the stated aim(s)? | Yes | A cross-sectional design is appropriate for assessing relationships between child, family, school, and neighborhood factors and flourishing. |
| 3. Was the sample size justified? | No information available | The study does not explicitly justify its sample size, though it includes 59,362 children from the National Survey of Children’s Health. |
| 4. Was the target/reference population clearly defined? | Yes | The study defines its population as children aged 6–17 years from the U.S. |
| 5. Was the sample frame appropriate? | Yes | The study uses data from the 2011–2012 National Survey of Children’s Health, ensuring national representativeness. |
| 6. Was the selection process representative? | Yes | The study uses weighted data to adjust for probability of selection and nonresponse bias. |
| 7. Were non-responders categorized? | No information available | No discussion of non-responders is provided. |
| 8. Were variables measured appropriately? | Yes | The study uses validated measures of flourishing, child development, and environmental influences. |
| 9. Were validated instruments used? | Yes | The study references established scales from previous research on child well-being. |
| 10. Was statistical significance clearly reported? | Yes | The study provides p-values, regression models, and confidence intervals. |
| 11. Were methods sufficiently described? | Yes | The study details its survey methodology, logistic regression approach, and weighting methods. |
| **Results** |  |  |
| 12. Were basic data adequately described? | Yes | The study presents descriptive statistics for key sociodemographic and behavioral variables. |
| 13. Does response rate raise concerns about bias? | No information available | The study does not explicitly state the response rate. |
| 14. Was information about non-responders described? | No | The study does not provide details on non-responders. |
| 15. Were the results internally consistent? | Yes | Findings align with the theoretical framework and prior literature. |
| 16. Were the results presented for all the analyses described in the methods? | Yes | The study presents findings for all planned analyses. |
| **Discussion** |  |  |
| 17. Were the authors' discussions and conclusions justified by the results? | Yes | The conclusions align with the reported findings and acknowledge study limitations. |
| 18. Were the limitations of the study discussed? | Yes | The study acknowledges its reliance on parent-reported data and potential recall bias. |
| **Other** |  |  |
| 19. Were there any funding sources or conflicts of interest? | Yes | The study acknowledges funding from the U.S. Department of Health and Human Services. |
| 20. Was ethical approval or consent of participants attained? | Yes | The study states that ethical approval was obtained, and informed consent was secured from participants. |
| 21. Was loss to follow-up after baseline 20% or less? | Not applicable | The study is cross-sectional and does not involve follow-up. |

**Table 19**

*Appraisal of Ditzel, Meiri, Casas, Ben-Arieh, and Torres-Vallejos (2023) using the AXIS Tool*

| **Question** | **Answer** | **Comments** |
| --- | --- | --- |
| **Introduction** |  |  |
| 1. Were the aims/objectives of the study clear? | Yes | The study examines satisfaction with the neighborhood and its effects on subjective well-being among Israeli and Chilean children. |
| **Methods** |  |  |
| 2. Was the study design appropriate for the stated aim(s)? | Yes | A cross-sectional design is appropriate for exploring the association between neighborhood satisfaction and well-being. |
| 3. Was the sample size justified? | No information available | The study does not explicitly justify its sample size, though it includes 4,942 children. |
| 4. Was the target/reference population clearly defined? | Yes | The study focuses on children aged 10 and 12 from Israel and Chile. |
| 5. Was the sample frame appropriate? | Yes | The study uses data from a structured survey conducted in both countries. |
| 6. Was the selection process representative? | No information available | The study does not specify how schools or children were selected. |
| 7. Were non-responders categorized? | No information available | No discussion of non-responders is provided. |
| 8. Were variables measured appropriately? | Yes | The study uses validated measures of subjective well-being and neighborhood satisfaction. |
| 9. Were validated instruments used? | Yes | The study references well-established subjective well-being scales. |
| 10. Was statistical significance clearly reported? | Yes | The study provides p-values and mediation model results. |
| 11. Were methods sufficiently described? | Yes | The study details its survey methodology and mediation analysis techniques. |
| **Results** |  |  |
| 12. Were basic data adequately described? | Yes | The study presents descriptive statistics and group comparisons. |
| 13. Does response rate raise concerns about bias? | Yes | The response rate is (36/94)*100% = 38%, which is lower than most studies. No information is available about bias in response. |
| 14. Was information about non-responders described? | No | The study does not provide details on non-responders. |
| 15. Were the results internally consistent? | Yes | Findings align with previous research on subjective well-being and neighborhood effects. |
| 16. Were the results presented for all the analyses described in the methods? | Yes | All planned analyses, including mediation effects, are reported. |
| **Discussion** |  |  |
| 17. Were the authors' discussions and conclusions justified by the results? | Yes | The conclusions are supported by the findings. |
| 18. Were the limitations of the study discussed? | Yes | The study acknowledges limitations such as cross-sectional design and cultural differences. |
| **Other** |  |  |
| 19. Were there any funding sources or conflicts of interest? | No information available | The study does not explicitly mention funding or conflicts of interest. |
| 20. Was ethical approval or consent of participants attained? | Yes | The study states that ethical approval was obtained, and informed consent was secured from participants. |
| 21. Was loss to follow-up after baseline 20% or less? | Not applicable | The study is cross-sectional and does not involve follow-up. |

**Table 20**

*Appraisal of Dardiry, Dimitrakaki, Tzavara, Ravens-Sieberer, and Tountas (2012) using the AXIS Tool*

| **Question** | **Answer** | **Comments** |
| --- | --- | --- |
| **Introduction** |  |  |
| 1. Were the aims/objectives of the study clear? | Yes | The study examines the relationship between socioeconomic status and children’s health-related quality of life in Greece. |
| **Methods** |  |  |
| 2. Was the study design appropriate for the stated aim(s)? | Yes | A cross-sectional design is appropriate for exploring associations between socioeconomic factors and health-related quality of life. |
| 3. Was the sample size justified? | No information available | The study does not explicitly justify its sample size, though it includes 1,194 children. |
| 4. Was the target/reference population clearly defined? | Yes | The study focuses on children aged 8–18 in Greece. |
| 5. Was the sample frame appropriate? | Yes | The study uses a structured school-based survey. |
| 6. Was the selection process representative? | No information available | The study does not clarify how schools were selected for participation. |
| 7. Were non-responders categorized? | No information available | No discussion of non-responders is provided. |
| 8. Were variables measured appropriately? | Yes | The study uses the KIDSCREEN-52 instrument, a validated measure of health-related quality of life. |
| 9. Were validated instruments used? | Yes | The KIDSCREEN-52 is a widely used, validated measure for child well-being. |
| 10. Was statistical significance clearly reported? | Yes | The study provides p-values and regression model results. |
| 11. Were methods sufficiently described? | Yes | The study details its survey methodology and statistical procedures. |
| **Results** |  |  |
| 12. Were basic data adequately described? | Yes | The study presents descriptive statistics and regression coefficients. |
| 13. Does response rate raise concerns about bias? | No | The response rates were 81.9% and 70.9%, which do not rate concerns about bias. |
| 14. Was information about non-responders described? | No | The study does not provide details on non-responders. |
| 15. Were the results internally consistent? | Yes | Findings align with previous research on socioeconomic status and health outcomes. |
| 16. Were the results presented for all the analyses described in the methods? | Yes | All planned analyses are reported. |
| **Discussion** |  |  |
| 17. Were the authors' discussions and conclusions justified by the results? | Yes | The conclusions are supported by the findings. |
| 18. Were the limitations of the study discussed? | Yes | The study acknowledges limitations such as cross-sectional design and self-report measures. |
| **Other** |  |  |
| 19. Were there any funding sources or conflicts of interest? | No information available | The study does not explicitly mention funding or conflicts of interest. |
| 20. Was ethical approval or consent of participants attained? | Yes | The study states that ethical approval was obtained, and informed consent was secured. |
| 21. Was loss to follow-up after baseline 20% or less? | Not applicable | The study is cross-sectional and does not involve follow-up. |

**Table 21**

*Appraisal of Forrester, Kahric, Lewis, and Rose (2024) using the AXIS Tool*

| **Question** | **Answer** | **Comments** |
| --- | --- | --- |
| **Introduction** |  |  |
| 1. Were the aims/objectives of the study clear? | Yes | The study investigates how neighborhood environment factors influence children’s social and emotional well-being. |
| **Methods** |  |  |
| 2. Was the study design appropriate for the stated aim(s)? | Yes | A cross-sectional design is appropriate for examining associations between neighborhood factors and child well-being. |
| 3. Was the sample size justified? | No information available | The study does not explicitly justify its sample size, though it includes 3,207 participants. |
| 4. Was the target/reference population clearly defined? | Yes | The study focuses on children aged 6–12 in urban and suburban neighborhoods. |
| 5. Was the sample frame appropriate? | Yes | The study uses a structured, school-based survey. |
| 6. Was the selection process representative? | No information available | The study does not clarify how schools were selected. |
| 7. Were non-responders categorized? | No information available | No discussion of non-responders is provided. |
| 8. Were variables measured appropriately? | Yes | The study uses validated measures for neighborhood perception and social-emotional well-being. |
| 9. Were validated instruments used? | Yes | The study references established well-being and neighborhood scales. |
| 10. Was statistical significance clearly reported? | Yes | The study provides p-values and regression model results. |
| 11. Were methods sufficiently described? | Yes | The study details its data collection and analysis methodology. |
| **Results** |  |  |
| 12. Were basic data adequately described? | Yes | The study presents descriptive statistics and neighborhood comparisons. |
| 13. Does response rate raise concerns about bias? | No information available | The response rate is not explicitly stated. |
| 14. Was information about non-responders described? | No | The study does not provide details on non-responders. |
| 15. Were the results internally consistent? | Yes | Findings align with previous research on neighborhood and child development. |
| 16. Were the results presented for all the analyses described in the methods? | Yes | All planned analyses, including subgroup comparisons, are reported. |
| **Discussion** |  |  |
| 17. Were the authors' discussions and conclusions justified by the results? | Yes | The conclusions align with the reported findings. |
| 18. Were the limitations of the study discussed? | Yes | The study acknowledges its reliance on self-reported data and cross-sectional limitations. |
| **Other** |  |  |
| 19. Were there any funding sources or conflicts of interest? | No information available | The study does not explicitly mention funding or conflicts of interest. |
| 20. Was ethical approval or consent of participants attained? | Yes | The study states that ethical approval was obtained, and informed consent was secured. |
| 21. Was loss to follow-up after baseline 20% or less? | Not applicable | The study is cross-sectional and does not involve follow-up. |

**Table 22**

*Appraisal of Alfaro, Benavente, Bedin, and Chuecas (2024) using the AXIS Tool*

| **Question** | **Answer** | **Comments** |
| --- | --- | --- |
| **Introduction** |  |  |
| 1. Were the aims/objectives of the study clear? | Yes | The study examines the relationship between life satisfaction and interpersonal relationships at family, school, and neighborhood levels for Chilean children. |
| **Methods** |  |  |
| 2. Was the study design appropriate for the stated aim(s)? | Yes | A cross-sectional design is appropriate for exploring the association between interpersonal relationships and life satisfaction. |
| 3. Was the sample size justified? | Yes | The study explicitly justifies its sample size. |
| 4. Was the target/reference population clearly defined? | Yes | The study focuses on children aged 10–12 in Chile. |
| 5. Was the sample frame appropriate? | Yes | The study uses a representative sample from the International Survey of Children’s Well-Being (ISCWeB). |
| 6. Was the selection process representative? | No information available | The study does not specify how participants were randomly selected. |
| 7. Were non-responders categorized? | No information available | No discussion of non-responders is provided. |
| 8. Were variables measured appropriately? | Yes | The study employs validated scales to measure life satisfaction and relationship satisfaction. |
| 9. Were validated instruments used? | Yes | The study references established instruments used in prior ISCWeB studies. |
| 10. Was statistical significance clearly reported? | Yes | The study provides p-values and structural equation modeling (SEM) results. |
| 11. Were methods sufficiently described? | Yes | The study details its SEM approach and variable measurements. |
| **Results** |  |  |
| 12. Were basic data adequately described? | Yes | The study presents descriptive statistics and model fit indices. |
| 13. Does response rate raise concerns about bias? | No | Response rates were 97% and 95.4%, which do not raise concerns about bias. |
| 14. Was information about non-responders described? | No | The study does not provide details on non-responders. |
| 15. Were the results internally consistent? | Yes | Findings align with theoretical frameworks and previous research. |
| 16. Were the results presented for all the analyses described in the methods? | Yes | All planned analyses, including mediation effects, are reported. |
| **Discussion** |  |  |
| 17. Were the authors' discussions and conclusions justified by the results? | Yes | The conclusions are supported by the findings. |
| 18. Were the limitations of the study discussed? | Yes | The study acknowledges limitations such as self-report bias and cultural specificity. |
| **Other** |  |  |
| 19. Were there any funding sources or conflicts of interest? | No information available | The study does not explicitly mention funding or conflicts of interest. |
| 20. Was ethical approval or consent of participants attained? | Yes | The study states that ethical approval was obtained, and informed consent was secured. |
| 21. Was loss to follow-up after baseline 20% or less? | Not applicable | The study is cross-sectional and does not involve follow-up. |

**Table 23**

*Appraisal of Ashiabi (2017) using the AXIS Tool*

| **Question** | **Answer** | **Comments** |
| --- | --- | --- |
| **Introduction** |  |  |
| 1. Were the aims/objectives of the study clear? | Yes | The study examines the impact of family economic hardship on children’s socioemotional development. |
| **Methods** |  |  |
| 2. Was the study design appropriate for the stated aim(s)? | Yes | A cross-sectional design is appropriate for assessing associations between economic hardship and child well-being. |
| 3. Was the sample size justified? | No information available | The study does not explicitly justify its sample size. |
| 4. Was the target/reference population clearly defined? | Yes | The study focuses on children from economically disadvantaged backgrounds in the U.S. |
| 5. Was the sample frame appropriate? | Yes | The study utilizes data from a large national survey. |
| 6. Was the selection process representative? | No information available | The study does not clarify the representativeness of its sample selection. |
| 7. Were non-responders categorized? | No information available | No discussion of non-responders is provided. |
| 8. Were variables measured appropriately? | Yes | The study employs validated measures of socioemotional development and economic hardship. |
| 9. Were validated instruments used? | Yes | The study references established scales for assessing child well-being. |
| 10. Was statistical significance clearly reported? | Yes | The study provides p-values and regression model results. |
| 11. Were methods sufficiently described? | Yes | The study details its data collection and statistical methodology. |
| **Results** |  |  |
| 12. Were basic data adequately described? | Yes | The study presents descriptive statistics and regression coefficients. |
| 13. Does response rate raise concerns about bias? | No information available | The response rate is not explicitly stated. |
| 14. Was information about non-responders described? | No | The study does not provide details on non-responders. |
| 15. Were the results internally consistent? | Yes | Findings align with theoretical expectations and prior research. |
| 16. Were the results presented for all the analyses described in the methods? | Yes | All planned analyses are reported. |
| **Discussion** |  |  |
| 17. Were the authors' discussions and conclusions justified by the results? | Yes | The conclusions align with the reported findings. |
| 18. Were the limitations of the study discussed? | Yes | The study acknowledges limitations such as self-report bias. |
| **Other** |  |  |
| 19. Were there any funding sources or conflicts of interest? | No information available | The study does not explicitly mention funding or conflicts of interest. |
| 20. Was ethical approval or consent of participants attained? | Yes | The study states that ethical approval was obtained, and informed consent was secured. |
| 21. Was loss to follow-up after baseline 20% or less? | Not applicable | The study is cross-sectional and does not involve follow-up. |

**References**

Downes, M. J., Brennan, M. L., Williams, H. C., & Dean, R. S. (2016). Development of a critical

appraisal tool to assess the quality of cross-sectional studies (AXIS). *BMJ open*, *6*(12), e011458.

National Institutes of Health (2014). *Quality Assessment Tool for Observational Cohort and*

*Cross-Sectional Studies*. Available online at: <https://www.nhlbi.nih.gov/health->

pro/guidelines/in-develop/cardiovascular-risk-reduction/tools/cohort (Accessed at 4 March 2025)

For questions:

Julia Steenwegen

Steenwegen@essb.eur.nl
