# Supplementary material for: The Neighborhood Resources that Shape Children’s Well-Being: A Systematic Literature Review
Source: Child Indic Res. 2026 Apr 13;19(4):1975–2013. doi: 10.1007/s12187-026-10362-x (PMC13570818; doi:10.1007/s12187-026-10362-x)
Supplement: Supplementary file 2 [file 12187_2026_10362_MOESM2_ESM.docx]

CASP Checklist:

For Qualitative Research

| **Reviewer Name:** | Julia Steenwegen |
| --- | --- |
| **Paper Title:** | Local belonging and ‘geographies of emotions’: Immigrant children’s experience of their neighbourhoods in Paris and Berlin |
| **Author:** | **Olga den Besten** |
| **Web Link:** |  |
| **Appraisal Date:** | April 2025 |

During critical appraisal, never make assumptions about what the researchers have done. If it is not possible to tell, use the “Can’t tell” response box. If you can’t tell, at best it means the researchers have not been explicit or transparent, but at worst it could mean the researchers have not undertaken a particular task or process. Once you’ve finished the critical appraisal, if there are a large number of “Can’t tell” responses, consider whether the findings of the study are trustworthy and interpret the results with caution.

| **Section A Are the results valid?** | |
| --- | --- |
| 1. Was there a clear statement of the aims of the research? | Yes |
| *CONSIDER:*   - *what was the goal of the research?* - *why was it thought important?* - *its relevance* | |
| 2. Is a qualitative methodology appropriate? | Yes |
| *CONSIDER:*   - *If the research seeks to interpret or illuminate the actions and/or subjective experiences of research participants* - *Is qualitative research the right methodology for addressing the research goal?* | |
| 3. Was the research design appropriate to address the aims of the research? | Yes |
| *CONSIDER:*   - *if the researcher has justified the research design (e.g., have they discussed how they decided which method to use)* | |
| 4. Was the recruitment strategy appropriate to the aims of the research? | Can’t Tell  **Limited detail on how children were selected or recruited.** |
| *CONSIDER:*   - *If the researcher has explained how the participants were selected* - *If they explained why the participants they selected were the most appropriate to provide access to the type of knowledge sought by the study* - *If there are any discussions around recruitment (e.g. why some people chose not to take part)* | |
| 5. Was the data collected in a way that addressed the research issue? | Yes |
| *CONSIDER:*   - *If the setting for the data collection was justified* - *If it is clear how data were collected (e.g. focus group, semi-structured interview etc.)* - *If the researcher has justified the methods chosen* - *If the researcher has made the methods explicit (e.g. for interview method, is there an indication of how interviews are conducted, or did they use a topic guide)* - *If methods were modified during the study. If so, has the researcher explained how and why* - *If the form of data is clear (e.g. tape recordings, video material, notes etc.)* - *If the researcher has discussed saturation of data* | |
| 6. Has the relationship between researcher and participants been adequately considered? | No  **No explicit reflection on researcher influence or power dynamics noted.** |
| *CONSIDER:*   - *If the researcher critically examined their own role, potential bias and influence during (a) formulation of the research questions (b) data collection, including sample recruitment and choice of location* - *How the researcher responded to events during the study and whether they considered the implications of any changes in the research design* | |
| **Section B: What are the results?** | |
| 7. Have ethical issues been taken into consideration? | Can’t Tell  **Ethics not directly discussed;** |
| *CONSIDER:*   - *If there are sufficient details of how the research was explained to participants for the reader to assess whether ethical standards were maintained* - *If the researcher has discussed issues raised by the study (e.g. issues around informed consent or confidentiality or how they have handled the effects of the study on the participants during and after the study)* - *If approval has been sought from the ethics committee* | |
| 8. Was the data analysis sufficiently rigorous? | Yes |
| *CONSIDER:*   - *If there is an in-depth description of the analysis process* - *If thematic analysis is used. If so, is it clear how the categories/themes were derived from the data* - *Whether the researcher explains how the data presented were selected from the original sample to demonstrate the analysis process* - *If sufficient data are presented to support the findings* - *To what extent contradictory data are taken into account* - *Whether the researcher critically examined their own role, potential bias and influence during analysis and selection of data for presentation* | |
| 9. Is there a clear statement of findings? | Yes |
| *CONSIDER:*   - *If the findings are explicit* - *If there is adequate discussion of the evidence both for and against the researcher’s arguments* - *If the researcher has discussed the credibility of their findings (e.g. triangulation, respondent validation, more than one analyst)* - *If the findings are discussed in relation to the original research question* | |
| **Section C: Will the results help locally?** | |
| 10. How valuable is the research? | Yes |
| *CONSIDER:*   - *If the researcher discusses the contribution the study makes to existing knowledge or understanding (e.g., do they consider the findings in relation to current practice or policy, or relevant research-based literature* - *If they identify new areas where research is necessary* - *If the researchers have discussed whether or how the findings can be transferred to other populations or considered other ways the research may be used* | |

| **APPRAISAL SUMMARY**: *List key points from your critical appraisal that need to be considered when assessing the validity of the results and their usefulness in decision-making.* | | |
| --- | --- | --- |
| **Positive/Methodologically sound** | **Negative/Relatively poor methodology** | **Unknowns** |
| **Clear aim and alignment of method and theory.**  **Child centred visual methods (subjective maps) enrich qualitative insights.** | **Lack of detail on participant recruitment and ethical protocols.**  **No mention of reflexivity or researcher positioning.** |  |

During critical appraisal, never make assumptions about what the researchers have done. If it is not possible to tell, use the “Can’t tell” response box. If you can’t tell, at best it means the researchers have not been explicit or transparent, but at worst it could mean the researchers have not undertaken a particular task or process. Once you’ve finished the critical appraisal, if there are a large number of “Can’t tell” responses, consider whether the findings of the study are trustworthy and interpret the results with caution.

| **Section A Are the results valid?** | |
| --- | --- |
| 11. Was there a clear statement of the aims of the research? | Yes  **Aim to explore children’s perspectives on health-promoting environments and the role of social capital is clearly stated** |
| *CONSIDER:*   - *what was the goal of the research?* - *why was it thought important?* - *its relevance* | |
| 12. Is a qualitative methodology appropriate? | Yes  **The study uses grounded theory and photovoice—appropriate for eliciting children’s lived experiences.** |
| *CONSIDER:*   - *If the research seeks to interpret or illuminate the actions and/or subjective experiences of research participants* - *Is qualitative research the right methodology for addressing the research goal?* | |
| 13. Was the research design appropriate to address the aims of the research? | Yes  **Combination of photovoice and grounded theory is a good fit for participatory and exploratory aims.** |
| *CONSIDER:*   - *if the researcher has justified the research design (e.g., have they discussed how they decided which method to use)* | |
| 14. Was the recruitment strategy appropriate to the aims of the research? | Yes  **Children from two neighborhoods with contrasting socio-economic status were included. Method of recruitment is explained** |
| *CONSIDER:*   - *If the researcher has explained how the participants were selected* - *If they explained why the participants they selected were the most appropriate to provide access to the type of knowledge sought by the study* - *If there are any discussions around recruitment (e.g. why some people chose not to take part)* | |
| 15. Was the data collected in a way that addressed the research issue? | Yes  **Participatory photography and discussions elicited rich data. Clear data collection process.** |
| *CONSIDER:*   - *If the setting for the data collection was justified* - *If it is clear how data were collected (e.g. focus group, semi-structured interview etc.)* - *If the researcher has justified the methods chosen* - *If the researcher has made the methods explicit (e.g. for interview method, is there an indication of how interviews are conducted, or did they use a topic guide)* - *If methods were modified during the study. If so, has the researcher explained how and why* - *If the form of data is clear (e.g. tape recordings, video material, notes etc.)* - *If the researcher has discussed saturation of data* | |
| 16. Has the relationship between researcher and participants been adequately considered? | Can’t Tell  **Reflexivity or researcher influence is not directly discussed, although participatory approach may imply consideration.** |
| *CONSIDER:*   - *If the researcher critically examined their own role, potential bias and influence during (a) formulation of the research questions (b) data collection, including sample recruitment and choice of location* - *How the researcher responded to events during the study and whether they considered the implications of any changes in the research design* | |
| **Section B: What are the results?** | |
| 17. Have ethical issues been taken into consideration? | Yes  **Ethical approval is mentioned; participatory research ethics are acknowledged.** |
| *CONSIDER:*   - *If there are sufficient details of how the research was explained to participants for the reader to assess whether ethical standards were maintained* - *If the researcher has discussed issues raised by the study (e.g. issues around informed consent or confidentiality or how they have handled the effects of the study on the participants during and after the study)* - *If approval has been sought from the ethics committee* | |
| 18. Was the data analysis sufficiently rigorous? | Yes  **Grounded theory approach described with coding stages; themes emerged from the data.** |
| *CONSIDER:*   - *If there is an in-depth description of the analysis process* - *If thematic analysis is used. If so, is it clear how the categories/themes were derived from the data* - *Whether the researcher explains how the data presented were selected from the original sample to demonstrate the analysis process* - *If sufficient data are presented to support the findings* - *To what extent contradictory data are taken into account* - *Whether the researcher critically examined their own role, potential bias and influence during analysis and selection of data for presentation* | |
| 19. Is there a clear statement of findings? | Yes |
| *CONSIDER:*   - *If the findings are explicit* - *If there is adequate discussion of the evidence both for and against the researcher’s arguments* - *If the researcher has discussed the credibility of their findings (e.g. triangulation, respondent validation, more than one analyst)* - *If the findings are discussed in relation to the original research question* | |
| **Section C: Will the results help locally?** | |
| 20. How valuable is the research? | Yes |
| *CONSIDER:*   - *If the researcher discusses the contribution the study makes to existing knowledge or understanding (e.g., do they consider the findings in relation to current practice or policy, or relevant research-based literature* - *If they identify new areas where research is necessary* - *If the researchers have discussed whether or how the findings can be transferred to other populations or considered other ways the research may be used* | |

| **APPRAISAL SUMMARY**: *List key points from your critical appraisal that need to be considered when assessing the validity of the results and their usefulness in decision-making.* | | |
| --- | --- | --- |
| **Positive/Methodologically sound** | **Negative/Relatively poor methodology** | **Unknowns** |
| **Clear aim and theory.**  **Strong participatory design and use of photovoice.**  **Rigorous analysis and rich findings.** | **Limited discussion of reflexivity and potential researcher bias.** |  |

During critical appraisal, never make assumptions about what the researchers have done. If it is not possible to tell, use the “Can’t tell” response box. If you can’t tell, at best it means the researchers have not been explicit or transparent, but at worst it could mean the researchers have not undertaken a particular task or process. Once you’ve finished the critical appraisal, if there are a large number of “Can’t tell” responses, consider whether the findings of the study are trustworthy and interpret the results with caution.

| **Section A Are the results valid?** | |
| --- | --- |
| 21. Was there a clear statement of the aims of the research? | Yes  **Aim is clearly to explore girls’ experiences of wellbeing, belonging, and their urban environments through participatory walking methods** |
| *CONSIDER:*   - *what was the goal of the research?* - *why was it thought important?* - *its relevance* | |
| 22. Is a qualitative methodology appropriate? | Yes  **The study is focused on children’s lived experiences: qualitative, participatory methodology is appropriate** |
| *CONSIDER:*   - *If the research seeks to interpret or illuminate the actions and/or subjective experiences of research participants* - *Is qualitative research the right methodology for addressing the research goal?* | |
| 23. Was the research design appropriate to address the aims of the research? | Yes  **The use of Participatory Photo Mapping and walking interviews aligns with the aim to engage girls in sharing local knowledge and experiences.** |
| *CONSIDER:*   - *if the researcher has justified the research design (e.g., have they discussed how they decided which method to use)* | |
| 24. Was the recruitment strategy appropriate to the aims of the research? | Yes  **39 girls aged 11–12 from a DEIS school were recruited over two years. The selection is clearly explained and relevant to the study’s context** |
| *CONSIDER:*   - *If the researcher has explained how the participants were selected* - *If they explained why the participants they selected were the most appropriate to provide access to the type of knowledge sought by the study* - *If there are any discussions around recruitment (e.g. why some people chose not to take part)* | |
| 25. Was the data collected in a way that addressed the research issue? | Yes  **Walking interviews, photo elicitation, mapping and narrative work all contributed rich, situated data.** |
| *CONSIDER:*   - *If the setting for the data collection was justified* - *If it is clear how data were collected (e.g. focus group, semi-structured interview etc.)* - *If the researcher has justified the methods chosen* - *If the researcher has made the methods explicit (e.g. for interview method, is there an indication of how interviews are conducted, or did they use a topic guide)* - *If methods were modified during the study. If so, has the researcher explained how and why* - *If the form of data is clear (e.g. tape recordings, video material, notes etc.)* - *If the researcher has discussed saturation of data* | |
| 26. Has the relationship between researcher and participants been adequately considered? | Yes  **There is reflexive discussion of power, child voice, and the value of walking methods in eliciting authentic experiences.** |
| *CONSIDER:*   - *If the researcher critically examined their own role, potential bias and influence during (a) formulation of the research questions (b) data collection, including sample recruitment and choice of location* - *How the researcher responded to events during the study and whether they considered the implications of any changes in the research design* | |
| **Section B: What are the results?** | |
| 27. Have ethical issues been taken into consideration? | Yes |
| *CONSIDER:*   - *If there are sufficient details of how the research was explained to participants for the reader to assess whether ethical standards were maintained* - *If the researcher has discussed issues raised by the study (e.g. issues around informed consent or confidentiality or how they have handled the effects of the study on the participants during and after the study)* - *If approval has been sought from the ethics committee* | |
| 28. Was the data analysis sufficiently rigorous? | ☐Yes |
| *CONSIDER:*   - *If there is an in-depth description of the analysis process* - *If thematic analysis is used. If so, is it clear how the categories/themes were derived from the data* - *Whether the researcher explains how the data presented were selected from the original sample to demonstrate the analysis process* - *If sufficient data are presented to support the findings* - *To what extent contradictory data are taken into account* - *Whether the researcher critically examined their own role, potential bias and influence during analysis and selection of data for presentation* | |
| 29. Is there a clear statement of findings? | Yes  **Findings highlight girls’ nuanced understandings of wellbeing, community, food access, safety, and dissonance with school health narratives.** |
| *CONSIDER:*   - *If the findings are explicit* - *If there is adequate discussion of the evidence both for and against the researcher’s arguments* - *If the researcher has discussed the credibility of their findings (e.g. triangulation, respondent validation, more than one analyst)* - *If the findings are discussed in relation to the original research question* | |
| **Section C: Will the results help locally?** | |
| 30. How valuable is the research? | Yes |
| *CONSIDER:*   - *If the researcher discusses the contribution the study makes to existing knowledge or understanding (e.g., do they consider the findings in relation to current practice or policy, or relevant research-based literature* - *If they identify new areas where research is necessary* - *If the researchers have discussed whether or how the findings can be transferred to other populations or considered other ways the research may be used* | |

| **APPRAISAL SUMMARY**: *List key points from your critical appraisal that need to be considered when assessing the validity of the results and their usefulness in decision-making.* | | |
| --- | --- | --- |
| **Positive/Methodologically sound** | **Negative/Relatively poor methodology** | **Unknowns** |
| **Participatory design rooted in child rights frameworks.**  **Multimodal methods suited to the aims.**  **Demonstrates clear understanding of context and ethics.** | **-** |  |

During critical appraisal, never make assumptions about what the researchers have done. If it is not possible to tell, use the “Can’t tell” response box. If you can’t tell, at best it means the researchers have not been explicit or transparent, but at worst it could mean the researchers have not undertaken a particular task or process. Once you’ve finished the critical appraisal, if there are a large number of “Can’t tell” responses, consider whether the findings of the study are trustworthy and interpret the results with caution.

| **Section A Are the results valid?** | |
| --- | --- |
| 31. Was there a clear statement of the aims of the research? | ☐Yes  Clear aim: to explore children's experiences of their neighbourhood, with focus on friendships, informal play, and well-being. |
| *CONSIDER:*   - *what was the goal of the research?* - *why was it thought important?* - *its relevance* | |
| 32. Is a qualitative methodology appropriate? | ☐Yes |
| *CONSIDER:*   - *If the research seeks to interpret or illuminate the actions and/or subjective experiences of research participants* - *Is qualitative research the right methodology for addressing the research goal?* | |
| 33. Was the research design appropriate to address the aims of the research? | ☐Yes  **The design involved participatory and ethnographic elements (e.g., drawings, mapping, interviews), well-matched to aims.** |
| *CONSIDER:*   - *if the researcher has justified the research design (e.g., have they discussed how they decided which method to use)* | |
| 34. Was the recruitment strategy appropriate to the aims of the research? | ☐Yes  **Children aged 7–12 in an urban disadvantaged Irish neighbourhood were purposively sampled.** |
| *CONSIDER:*   - *If the researcher has explained how the participants were selected* - *If they explained why the participants they selected were the most appropriate to provide access to the type of knowledge sought by the study* - *If there are any discussions around recruitment (e.g. why some people chose not to take part)* | |
| 35. Was the data collected in a way that addressed the research issue? | ☐Yes  **A wide range of methods (including visual tools) were used to allow children to articulate experiences beyond verbal accounts.** |
| *CONSIDER:*   - *If the setting for the data collection was justified* - *If it is clear how data were collected (e.g. focus group, semi-structured interview etc.)* - *If the researcher has justified the methods chosen* - *If the researcher has made the methods explicit (e.g. for interview method, is there an indication of how interviews are conducted, or did they use a topic guide)* - *If methods were modified during the study. If so, has the researcher explained how and why* - *If the form of data is clear (e.g. tape recordings, video material, notes etc.)* - *If the researcher has discussed saturation of data* | |
| 36. Has the relationship between researcher and participants been adequately considered? | ☐Yes  **The paper discusses child-centred approaches, respect for children’s knowledge, and inclusive, empowering methods.** |
| *CONSIDER:*   - *If the researcher critically examined their own role, potential bias and influence during (a) formulation of the research questions (b) data collection, including sample recruitment and choice of location* - *How the researcher responded to events during the study and whether they considered the implications of any changes in the research design* | |
| **Section B: What are the results?** | |
| 37. Have ethical issues been taken into consideration? | ☐Yes |
| *CONSIDER:*   - *If there are sufficient details of how the research was explained to participants for the reader to assess whether ethical standards were maintained* - *If the researcher has discussed issues raised by the study (e.g. issues around informed consent or confidentiality or how they have handled the effects of the study on the participants during and after the study)* - *If approval has been sought from the ethics committee* | |
| 38. Was the data analysis sufficiently rigorous? | ☐Yes  **Themes were drawn from detailed qualitative material. The analysis is supported with illustrative quotes and connected to literature.** |
| *CONSIDER:*   - *If there is an in-depth description of the analysis process* - *If thematic analysis is used. If so, is it clear how the categories/themes were derived from the data* - *Whether the researcher explains how the data presented were selected from the original sample to demonstrate the analysis process* - *If sufficient data are presented to support the findings* - *To what extent contradictory data are taken into account* - *Whether the researcher critically examined their own role, potential bias and influence during analysis and selection of data for presentation* | |
| 39. Is there a clear statement of findings? | ☐Yes  **Strong focus on the value of friendships, autonomy in neighbourhood space, and children's agency is clearly conveyed.** |
| *CONSIDER:*   - *If the findings are explicit* - *If there is adequate discussion of the evidence both for and against the researcher’s arguments* - *If the researcher has discussed the credibility of their findings (e.g. triangulation, respondent validation, more than one analyst)* - *If the findings are discussed in relation to the original research question* | |
| **Section C: Will the results help locally?** | |
| 40. How valuable is the research? | ☐Yes |
| *CONSIDER:*   - *If the researcher discusses the contribution the study makes to existing knowledge or understanding (e.g., do they consider the findings in relation to current practice or policy, or relevant research-based literature* - *If they identify new areas where research is necessary* - *If the researchers have discussed whether or how the findings can be transferred to other populations or considered other ways the research may be used* | |

| **APPRAISAL SUMMARY**: *List key points from your critical appraisal that need to be considered when assessing the validity of the results and their usefulness in decision-making.* | | |
| --- | --- | --- |
| **Positive/Methodologically sound** | **Negative/Relatively poor methodology** | **Unknowns** |
| **Deeply grounded in children’s own perspectives.**  **Strong alignment of aims, design, and participatory methods.**  **Clearly communicated and relevant findings.** | **-** |  |

During critical appraisal, never make assumptions about what the researchers have done. If it is not possible to tell, use the “Can’t tell” response box. If you can’t tell, at best it means the researchers have not been explicit or transparent, but at worst it could mean the researchers have not undertaken a particular task or process. Once you’ve finished the critical appraisal, if there are a large number of “Can’t tell” responses, consider whether the findings of the study are trustworthy and interpret the results with caution.

| **Section A Are the results valid?** | |
| --- | --- |
| 41. Was there a clear statement of the aims of the research? | **Yes** The study clearly aimed to explore how children living in disadvantaged areas perceive their communities and actively manage threats to their safety and wellbeing. |
| *CONSIDER:*   - *what was the goal of the research?* - *why was it thought important?* - *its relevance* | |
| 42. Is a qualitative methodology appropriate? | **Yes** The research sought to understand children’s subjective experiences and coping strategies |
| *CONSIDER:*   - *If the research seeks to interpret or illuminate the actions and/or subjective experiences of research participants* - *Is qualitative research the right methodology for addressing the research goal?* | |
| 43. Was the research design appropriate to address the aims of the research? | **Yes** The study used interviews and discussion groups with children aged 8–14 from four disadvantaged communities |
| *CONSIDER:*   - *if the researcher has justified the research design (e.g., have they discussed how they decided which method to use)* | |
| 44. Was the recruitment strategy appropriate to the aims of the research? | **Yes** Children were recruited through schools and a parallel parent study. The sample included various ages and both genders across multiple settings, ensuring a diverse representation of experiences. |
| *CONSIDER:*   - *If the researcher has explained how the participants were selected* - *If they explained why the participants they selected were the most appropriate to provide access to the type of knowledge sought by the study* - *If there are any discussions around recruitment (e.g. why some people chose not to take part)* | |
| 45. Was the data collected in a way that addressed the research issue? | ☐ **Yes** Semi-structured interviews and focus groups |
| *CONSIDER:*   - *If the setting for the data collection was justified* - *If it is clear how data were collected (e.g. focus group, semi-structured interview etc.)* - *If the researcher has justified the methods chosen* - *If the researcher has made the methods explicit (e.g. for interview method, is there an indication of how interviews are conducted, or did they use a topic guide)* - *If methods were modified during the study. If so, has the researcher explained how and why* - *If the form of data is clear (e.g. tape recordings, video material, notes etc.)* - *If the researcher has discussed saturation of data* | |
| 46. Has the relationship between researcher and participants been adequately considered? | **Can’t tell** While interviews were conducted sensitively and in familiar settings (schools or homes), the authors do not explicitly reflect on their own roles, potential biases, or positionality. |
| *CONSIDER:*   - *If the researcher critically examined their own role, potential bias and influence during (a) formulation of the research questions (b) data collection, including sample recruitment and choice of location* - *How the researcher responded to events during the study and whether they considered the implications of any changes in the research design* | |
| **Section B: What are the results?** | |
| 47. Have ethical issues been taken into consideration? | **Yes** Parental and child consent was obtained, and children were interviewed in safe environments. Confidentiality and assent were respected. |
| *CONSIDER:*   - *If there are sufficient details of how the research was explained to participants for the reader to assess whether ethical standards were maintained* - *If the researcher has discussed issues raised by the study (e.g. issues around informed consent or confidentiality or how they have handled the effects of the study on the participants during and after the study)* - *If approval has been sought from the ethics committee* | |
| 48. Was the data analysis sufficiently rigorous? | **Yes** Thematic coding using NVivo was described. Themes were developed both inductively and deductively. Transcripts were analysed for age, gender, and area differences, though no mention was made of member checking or multiple coders |
| *CONSIDER:*   - *If there is an in-depth description of the analysis process* - *If thematic analysis is used. If so, is it clear how the categories/themes were derived from the data* - *Whether the researcher explains how the data presented were selected from the original sample to demonstrate the analysis process* - *If sufficient data are presented to support the findings* - *To what extent contradictory data are taken into account* - *Whether the researcher critically examined their own role, potential bias and influence during analysis and selection of data for presentation* | |
| 49. Is there a clear statement of findings? | **Yes** Findings are structured around thematic insights |
| *CONSIDER:*   - *If the findings are explicit* - *If there is adequate discussion of the evidence both for and against the researcher’s arguments* - *If the researcher has discussed the credibility of their findings (e.g. triangulation, respondent validation, more than one analyst)* - *If the findings are discussed in relation to the original research question* | |
| **Section C: Will the results help locally?** | |
| 50. How valuable is the research? | **Yes** This study makes an important contribution by centering children’s voices on community risk and resilience. |
| *CONSIDER:*   - *If the researcher discusses the contribution the study makes to existing knowledge or understanding (e.g., do they consider the findings in relation to current practice or policy, or relevant research-based literature* - *If they identify new areas where research is necessary* - *If the researchers have discussed whether or how the findings can be transferred to other populations or considered other ways the research may be used* | |

| **APPRAISAL SUMMARY**: *List key points from your critical appraisal that need to be considered when assessing the validity of the results and their usefulness in decision-making.* | | |
| --- | --- | --- |
| **Positive/Methodologically sound** | **Negative/Relatively poor methodology** | **Unknowns** |
| 1. Clear aim and rationale grounded in resilience and risk theory 2. Strong use of interviews and focus groups with diverse participants 3. Rich, thematic analysis with attention to age, gender, and area differences 4. Concrete, actionable implications for practice and policy | 1. Researcher reflexivity is not explicitly addressed 2. No mention of respondent validation or inter-coder reliability |  |

During critical appraisal, never make assumptions about what the researchers have done. If it is not possible to tell, use the “Can’t tell” response box. If you can’t tell, at best it means the researchers have not been explicit or transparent, but at worst it could mean the researchers have not undertaken a particular task or process. Once you’ve finished the critical appraisal, if there are a large number of “Can’t tell” responses, consider whether the findings of the study are trustworthy and interpret the results with caution.

| **Section A Are the results valid?** | |
| --- | --- |
| 51. Was there a clear statement of the aims of the research? | **Yes** The study aimed to explore how the home and neighbourhood environments, and their interaction, affect child wellbeing in two high-poverty areas in England. |
| *CONSIDER:*   - *what was the goal of the research?* - *why was it thought important?* - *its relevance* | |
| 52. Is a qualitative methodology appropriate? | **Yes** The study focused on lived experiences and perceptions |
| *CONSIDER:*   - *If the research seeks to interpret or illuminate the actions and/or subjective experiences of research participants* - *Is qualitative research the right methodology for addressing the research goal?* | |
| 53. Was the research design appropriate to address the aims of the research? | **Yes** The study used semi-structured interviews, photos, and thematic analysis to explore interactions between housing and neighbourhood environments. |
| *CONSIDER:*   - *if the researcher has justified the research design (e.g., have they discussed how they decided which method to use)* | |
| 54. Was the recruitment strategy appropriate to the aims of the research? | ☐Yes |
| *CONSIDER:*   - *If the researcher has explained how the participants were selected* - *If they explained why the participants they selected were the most appropriate to provide access to the type of knowledge sought by the study* - *If there are any discussions around recruitment (e.g. why some people chose not to take part)* | |
| 55. Was the data collected in a way that addressed the research issue? | **Yes** Semi-structured interviews, optional photo elicitation, and a supporting questionnaire (HAPIE tool) allowed for comprehensive and relevant data collection. |
| *CONSIDER:*   - *If the setting for the data collection was justified* - *If it is clear how data were collected (e.g. focus group, semi-structured interview etc.)* - *If the researcher has justified the methods chosen* - *If the researcher has made the methods explicit (e.g. for interview method, is there an indication of how interviews are conducted, or did they use a topic guide)* - *If methods were modified during the study. If so, has the researcher explained how and why* - *If the form of data is clear (e.g. tape recordings, video material, notes etc.)* - *If the researcher has discussed saturation of data* | |
| 56. Has the relationship between researcher and participants been adequately considered? | **Yes** Interviews were conducted by community researchers who shared social proximity with participants. Reflexivity is implicit in the participatory design, though more explicit discussion would strengthen this. |
| *CONSIDER:*   - *If the researcher critically examined their own role, potential bias and influence during (a) formulation of the research questions (b) data collection, including sample recruitment and choice of location* - *How the researcher responded to events during the study and whether they considered the implications of any changes in the research design* | |
| **Section B: What are the results?** | |
| 57. Have ethical issues been taken into consideration? | ☐**Yes** Ethical procedures are outlined, including verbal consent, confidentiality, and accommodations for digital or language barriers. |
| *CONSIDER:*   - *If there are sufficient details of how the research was explained to participants for the reader to assess whether ethical standards were maintained* - *If the researcher has discussed issues raised by the study (e.g. issues around informed consent or confidentiality or how they have handled the effects of the study on the participants during and after the study)* - *If approval has been sought from the ethics committee* | |
| 58. Was the data analysis sufficiently rigorous? | **Yes** Thematic analysis was used with double coding, use of NVivo, and iteration across two sites. Rich quotes support findings. However, member checking or triangulation are not reported. |
| *CONSIDER:*   - *If there is an in-depth description of the analysis process* - *If thematic analysis is used. If so, is it clear how the categories/themes were derived from the data* - *Whether the researcher explains how the data presented were selected from the original sample to demonstrate the analysis process* - *If sufficient data are presented to support the findings* - *To what extent contradictory data are taken into account* - *Whether the researcher critically examined their own role, potential bias and influence during analysis and selection of data for presentation* | |
| 59. Is there a clear statement of findings? | **Yes** Ten themes are clearly presented, with illustrative quotes, figures, and cross-site comparison. Findings are contextualised within existing literature and policy. |
| *CONSIDER:*   - *If the findings are explicit* - *If there is adequate discussion of the evidence both for and against the researcher’s arguments* - *If the researcher has discussed the credibility of their findings (e.g. triangulation, respondent validation, more than one analyst)* - *If the findings are discussed in relation to the original research question* | |
| **Section C: Will the results help locally?** | |
| 60. How valuable is the research? | **Yes** This study offers timely and rich insights for urban planners, housing policy makers, and public health officials. It shows the compound effects of poor housing and local area neglect on children’s wellbeing, and identifies specific opportunities for intervention. |
| *CONSIDER:*   - *If the researcher discusses the contribution the study makes to existing knowledge or understanding (e.g., do they consider the findings in relation to current practice or policy, or relevant research-based literature* - *If they identify new areas where research is necessary* - *If the researchers have discussed whether or how the findings can be transferred to other populations or considered other ways the research may be used* | |

| **APPRAISAL SUMMARY**: *List key points from your critical appraisal that need to be considered when assessing the validity of the results and their usefulness in decision-making.* | | |
| --- | --- | --- |
| **Positive/Methodologically sound** | **Negative/Relatively poor methodology** | **Unknowns** |
| 1. Clear and timely research aim 2. Co-produced design with community researchers 3. Purposeful inclusion of seldom-heard populations 4. Thematic analysis with strong thematic coherence 5. Useful for informing local-level policy and urban design | Limited discussion of researcher reflexivity |  |

**Referencing recommendation:**

CASP recommends using the Harvard style referencing, which is an author/date method. Sources are cited within the body of your assignment by giving the name of the author(s) followed by the date of publication. All other details about the publication are given in the list of references or bibliography at the end.

Example:

*Critical Appraisal Skills Programme (2024). CASP (insert name of checklist i.e. systematic reviews with meta-analysis of randomised controlled trials (RCTs) Checklist.) [online] Available at: insert URL. Accessed: insert date accessed.*

**Creative Commons**

©CASP this work is licensed under the Creative Commons Attribution – Non-Commercial- Share A like. To view a copy of this licence, visit <https://creativecommons.org/licenses/by-nc-sa/4.0/>

**Need further training on evidence-based decision making?** Our online training courses are helpful for healthcare educational researchers and any other learners who:

- Need to critically appraise and stay abreast of the healthcare research literature as part of their clinical duties.
- Are considering carrying out research & developing their own research projects.
- Make decisions in their role, whether that be policy making or patient facing.

**Benefits of CASP Training:**

- Affordable – courses start from as little as £6
- Professional training – leading experts in critical appraisal training
- Self-directed study – complete each course in your own time
- 12 months access – revisit areas you aren’t sure of and revise
- CPD certification - after each completed module

Scan the QR code below or visit <https://casp-uk.net/critical-appraisal-online-training-courses/> for more information and to start learning more.

For questions:

Julia Steenwegen

Steenwegen@essb.eur.nl
